# HFNet: A CNN Architecture Co-designed for Neuromorphic Hardware With a Crossbar Array of Synapses

**DOI:** 10.3389/fnins.2020.00907

**Published:** 2020-10-26

**Authors:** Roshan Gopalakrishnan, Yansong Chua, Pengfei Sun, Ashish Jith Sreejith Kumar, Arindam Basu

**Affiliations:** ^1^Institute for Infocomm Research, Agency for Science, Technology and Research (ASTAR), Singapore, Singapore; ^2^School of Electrical and Electronic Engineering, Nanyang Technological University, Singapore, Singapore

**Keywords:** neuromorphic computing, neuromorphic chip, hardware constraints, deep learning, neural network, crossbar array, convolution, convolutional neural network

## Abstract

The hardware-software co-optimization of neural network architectures is a field of research that emerged with the advent of commercial neuromorphic chips, such as the IBM TrueNorth and Intel Loihi. Development of simulation and automated mapping software tools in tandem with the design of neuromorphic hardware, whilst taking into consideration the hardware constraints, will play an increasingly significant role in deployment of system-level applications. This paper illustrates the importance and benefits of co-design of convolutional neural networks (CNN) that are to be mapped onto neuromorphic hardware with a crossbar array of synapses. Toward this end, we first study which convolution techniques are more hardware friendly and propose different mapping techniques for different convolutions. We show that, for a seven-layered CNN, our proposed mapping technique can reduce the number of cores used by 4.9–13.8 times for crossbar sizes ranging from 128 × 256 to 1,024 × 1,024, and this can be compared to the toeplitz method of mapping. We next develop an iterative co-design process for the systematic design of more hardware-friendly CNNs whilst considering hardware constraints, such as core sizes. A python wrapper, developed for the mapping process, is also useful for validating hardware design and studies on traffic volume and energy consumption. Finally, a new neural network dubbed HFNet is proposed using the above co-design process; it achieves a classification accuracy of 71.3% on the IMAGENET dataset (comparable to the VGG-16) but uses 11 times less cores for neuromorphic hardware with core size of 1,024 × 1,024. We also modified the HFNet to fit onto different core sizes and report on the corresponding classification accuracies. Various aspects of the paper are patent pending.

## 1. Introduction

Over the past decade, GPUs have emerged as a major hardware resource for deep learning tasks. However, fields, such as the internet of things (IoT) and edge computing are constantly in need of more efficient neural-network-specific hardware (Basu et al., 2018; Deng et al., 2018; Alyamkin et al., 2019; Roy et al., 2019). This encourages competition among companies, such as Intel, IBM, and others to propose new hardware alternatives, leading to the emergence of commercially available deep learning accelerators (Barry et al., 2015; Jouppi et al., 2017) and neuromorphic chips (Esser et al., 2016; Davies et al., 2018; Pei et al., 2019). Deep learning accelerators are application specific integrated circuits (ASICs) tailored for artificial neural networks (ANN), whereas, neuromorphic chips can fall in two categories (Bose et al., 2019): (1) ASICs with biologically inspired spiking neural networks (SNN), which contain networks of neurons and synapses for computation and communication, or (2) ASICs with analog computing by exploiting dense non-volatile memory based crossbars to accelerate matrix-vector multiplications. Our paper is not concerned with any specific hardware but any neuromorphic architecture relying on analog crossbars for matrix-vector multiplications.

**Figure 1:**
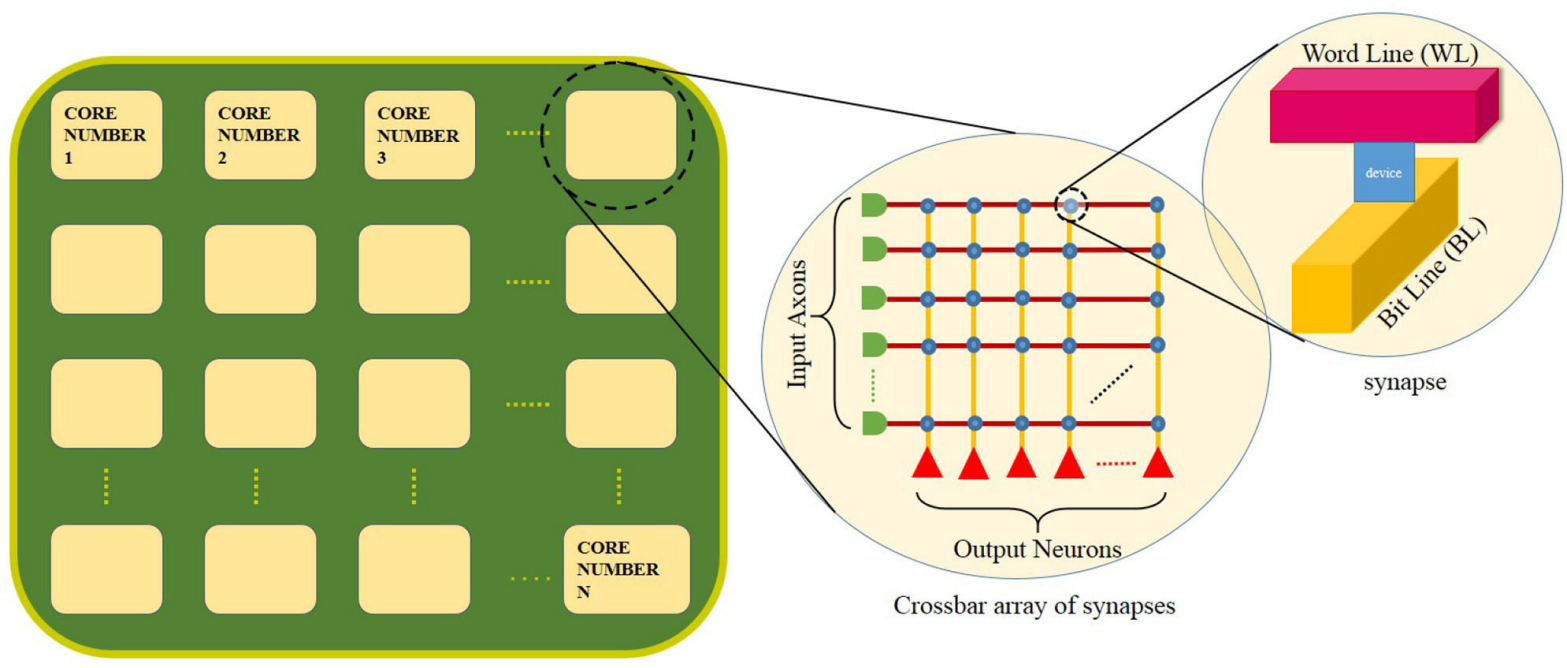
A schematic of a neuromorphic chip with N number of neuromorphic cores. The first inset shows the crossbar array of synapses within each core. A memory device is used to implement each synapse at the crossbar intersection (as shown in the second inset).

A schematic of a generic crossbar-based neuromorphic chip is shown in Figure 1. The chip has “N” number of neuromorphic cores. Network on chip (NoC) or router interfaces are not shown for illustration purposes. Each neuromorphic core contains a crossbar array of synapses as shown in the first inset of the figure. The rows and columns of the crossbar correspond to input axons and output neurons, respectively. These axons and neurons are interconnected to each other and represented in the form of blue dots at these intersections. Within each intersection of the crossbar between the word line and the bit line is a synaptic device that has memory and can perform in-memory computation (as shown in the second inset). The crossbar structure is well-suited for performing matrix-vector multiplication (MVM) (Hu et al., 2016) along each column in a crossbar architecture. For instance, a neuromorphic core with a core size of 256 × 256, input voltages from the respective 256 axons are fed through each word line (red horizontal lines). The bit line (yellow vertical lines) collects all of the weighted current at each synaptic node (256 × 256) and delivers to the respective output neurons for integration. The neuromorphic core size refers to the number of axons (axon size, AS) × the number of neurons (neuron size, NS) in a single neuromorphic core. The weighted current depends on the memory element at each intersection of the word line and bit line. In analog devices, using Kirchoff's current law, the total current flowing into each neuron from the respective bit lines is the sum of currents flowing through each intersection in every column. This corresponds well with how inputs in a neural network is the weighted sum of input voltages (∑(*Input* × *Weight*)). Considering such a neuromorphic chip, there are several hardware constraints: firstly, at the single device, we may have low bit precision of synaptic weights and output activations (Ji et al., 2018; Deng et al., 2020), synaptic noise and variability (Ambrogio et al., 2014a,b). Secondly, in the chip architecture, we have a limited number of neuromorphic cores and a limitation in the core size of each neuromorphic core and the fan-in/fan-out degree of each neuron (Ji et al., 2018; Gopalakrishnan et al., 2019b).

The neuromorphic chip considered in this paper is based on a crossbar architecture (Prezioso et al., 2015) of non-volatile memory synapses. Crossbar architecture fits well for fully connected neural networks, such as the multi-layered perceptron (MLP). Given that one of the popularly used layers in SNNs are fully connected ones (Diehl and Cook, 2015), crossbar architectures are a natural fit. However, with recent advancement in research related to conversion of ANNs to SNNs (Rueckauer et al., 2016) and training of convolutional SNNs (Wu et al., 2019), one of the main challenges is to efficiently map the neurons in a CNN onto the neuromorphic chip while fulfilling hardware constraints, such as core size, number of cores and fan-in/fan-out (Ji et al., 2016). Given existing CNNs and neuromorphic hardwares, how can we best map the CNN onto the hardware using the least number of cores? This requires understanding of the computation at each crossbar array and how best to map each convolutional layer onto the core. We may then ask what convolution layers are best suited for mapping onto neuromorphic hardwares. This is the first major contribution of this paper and these questions are addressed under section Mapping. Existing neuromorphic chips have a mapping framework which is hardware specific. IBM TrueNorth (Akopyan et al., 2015) uses Matlab based Corelet (Amir et al., 2013) which is specific to their hardware. Within Corelet, a mapping technique is implemented as a minimization problem (Akopyan et al., 2015). SpiNNaker and BrainScaleS use a simulator-independent Python wrapper, PyNN (Andrew et al., 2009). Sequential mapping is used in SpiNNaker while neural engineering framework (NEF) is used for Neurogrid (Voelker et al., 2017). Neutrams (Ji et al., 2016) implements an optimized mapping technique based on the graph partition problem: Kernighan-Lin (KL) partitioning strategy for network on chip (NoC). For mapping CNNs onto neuromorphic hardwares, an iterative process is implemented using a Python wrapper, which is also discussed in section 2.3.2.

While developing deep neural networks that are to be mapped onto a neuromorphic chip, one need not in principle be aware of the underlying hardware architecture. The mapping above assumes that the CNNs and hardware constraints are given. We may however further ask how software and hardware co-design can give us both CNNs and neuromorphic hardwares that are mapped using fewer cores while achieving similar classification accuracies. Specifically, given a neuromophic hardware with square crossbar array, we would like to design a CNN that utilizes fewer cores (section Co-design). In this regard, one may take two approaches, either design the network from scratch so as to satisfy the hardware constraints (Esser et al., 2016) or modify an existing CNN, such as reducing the number of features (feature maps) in each convolution layer without having to split the convolution matrix among different cores (“core matrix splitting”) whereby axons and weight matrix of a particular layer are split onto multiple cores (detailed in subsection 2.2.1). This is the second major contribution of this paper, and the proposed novel hardware-friendly CNN, HFNet, is obtained by iteratively modifying the layers of existing CNNs (VGG, MobileNet, NIN, and Squeezenet; this is discussed in section 2) and the number of features (feature maps) in some layers are altered so as to fit onto cores of different sizes. This is done to avoid core matrix splitting. Finally, the different versions of HFNet are trained and their classification accuracies on the IMAGENET dataset (Deng et al., 2009) tested so as to study the trade-off between accuracies and core sizes (section 3.2). This work is mainly focused on mapping of feedforward deep neural networks (DNN). During the investigation of mapping techniques, we understood that designing and mapping of a CNN must be performed in close relation to each other for better hardware utilization. Hence, as a beginning, we have limited the design space to just feedforward networks instead of skipped connections. We have considered the better-performing feedforward CNN MobileNet as an initial candidate for mapping and later modified to HFNet based on mapping and traditional deep learning techniques.

The trained CNNs have full precision weights and activation values for fair comparison with existing CNNs. Hardware limitations on synapses, such as low precision weights and variability issues are not within the scope of our work.

The paper is organized as follows. Section 2 mainly contains two subsections, one on mapping and another on co-design. Mapping describes the computation and mapping in a crossbar array. Co-design is illustrated with the issue of core matrix splitting and then the motivation and design flow of the proposed hardware-friendly neural network, HFNet. Section 3 provides an experimental framework for the two subsections, mapping and co-design in section 2. The classification accuracy of different versions of HFNet on the IMAGENET dataset is included here. The paper is concluded in section 4 with a discussion of future works.

## 2. Materials and Methods

### 2.1. Mapping

#### 2.1.1. Computation in a Crossbar Architecture

**Figure 2:**
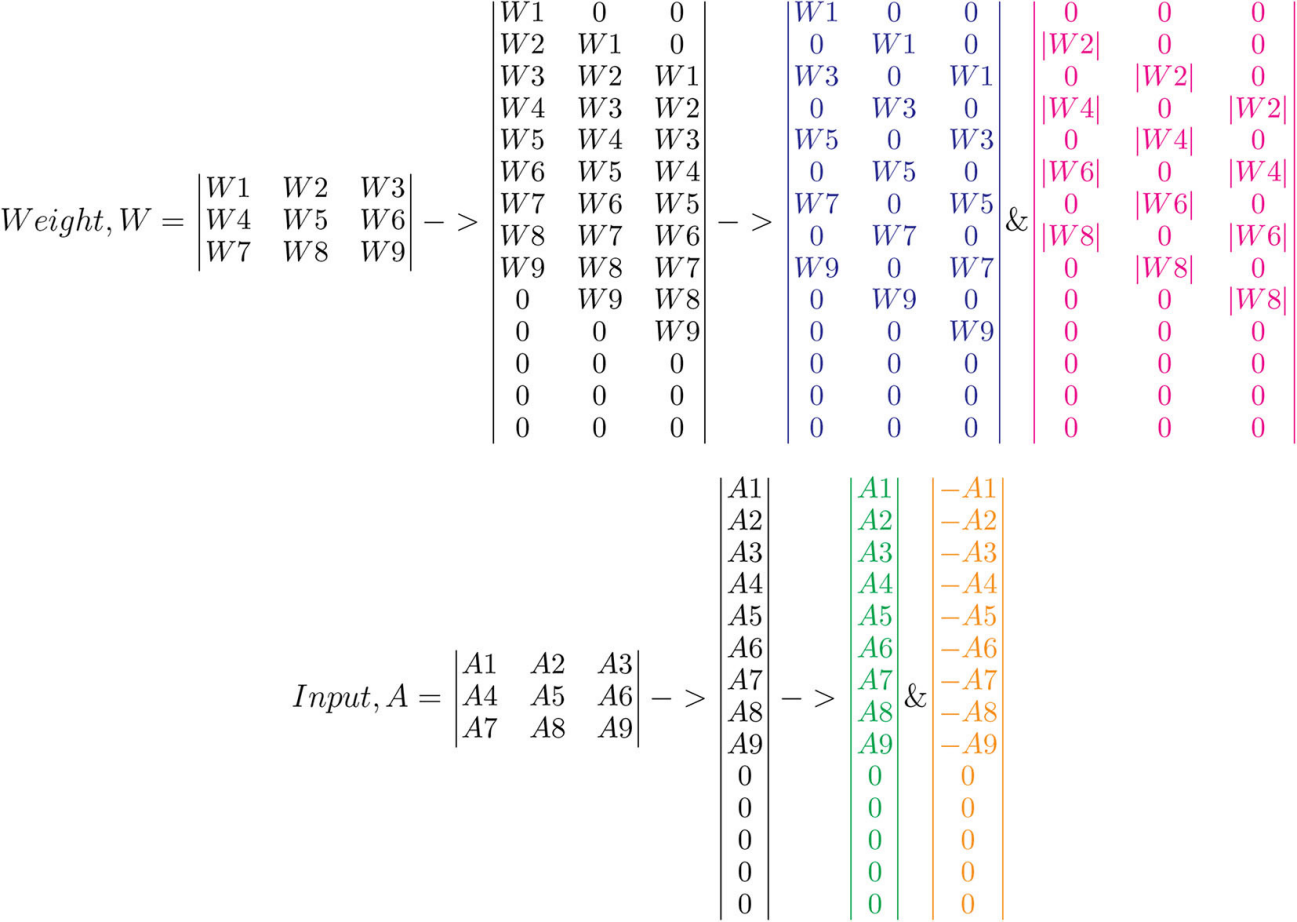
Division of network parameters–weights and input activations into positive and negative matrices. Here, W2, W4, W6, and W8 are negative, whereas the remaining weights are positive. Note that the text color codes are in correspondence to the color codes used in Figure 3. Adapted from Gopalakrishnan et al. (2019a).

The crossbar array of synapses in a neuromorphic chip can be used to perform convolutions. Mathematically, convolution is the sum of dot product of feature and input matrices (Figure 2). In CNNs, the input matrix will be the activations from the prior layer while the filter matrix is the convolution filter kernel, saved as weights, W after training. Since these weights can be either positive or negative, one way of implementing convolution on a crossbar array is to split the weights into positive and negative matrices along with two copies of input matrices in positive and negative values. The details of the matrix generation is shown in Figure 2, which incorporate the convolution operation in crossbar arrays as described in (Yakopcic et al., 2016). A single column crossbar gives the output of an element of the convolution operation, which is provided to the corresponding neuron. Convolution operation is extended across multiple columns of synapses to be computed in parallel. This requires the weights and inputs to be represented in a toeplitz matrix, as shown in Figure 2 (Yakopcic et al., 2017), Figure 3 illustrated such an implementation. This implementation doubles the hardware resources required, which is also the case in IBM Truenorth (Esser et al., 2016), where two synapses are required to implement the ternary weights −1, 0, +1. IBM Truenorth also uses toeplitz structure or structured kernels for mapping (Appuswamy et al., 2016). In order to mitigate the aforementioned doubling of hardware requirement in a neuromorphic hardware, one can implement two memory devices at each synapse to represent both the positive and negative weights by subtraction. This implementation does not need two copies of weights; generating a single weight toeplitz matrix is sufficient.

**Figure 3:**
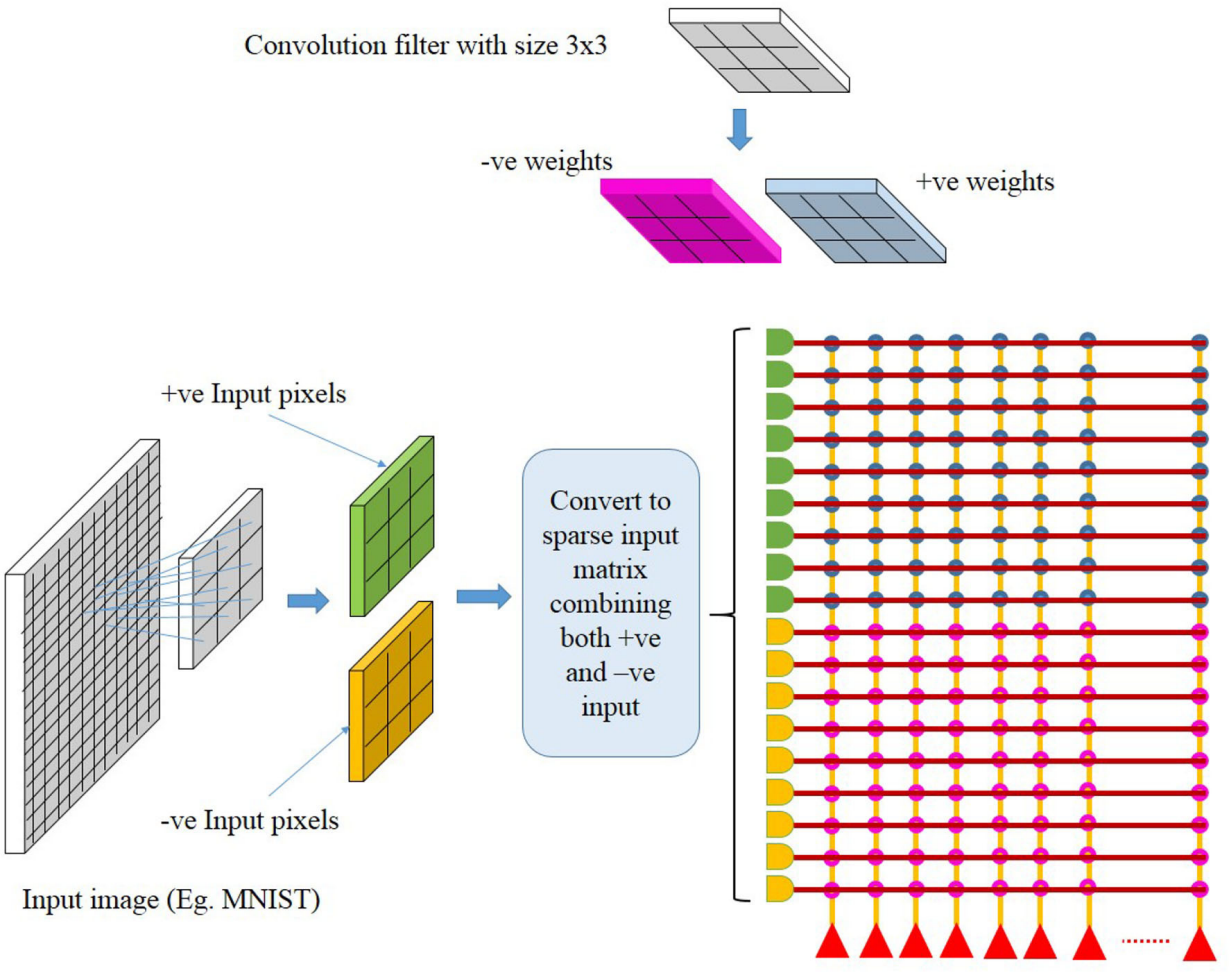
Computation in a crossbar architecture within a single neuromorphic core. Follow the color codes representing the partitioning of input and weight matrices and its corresponding mapping onto the crossbar array. Taken from Gopalakrishnan et al. (2019a).

#### 2.1.2. Mapping on a Crossbar Architecture

**Figure 4:**
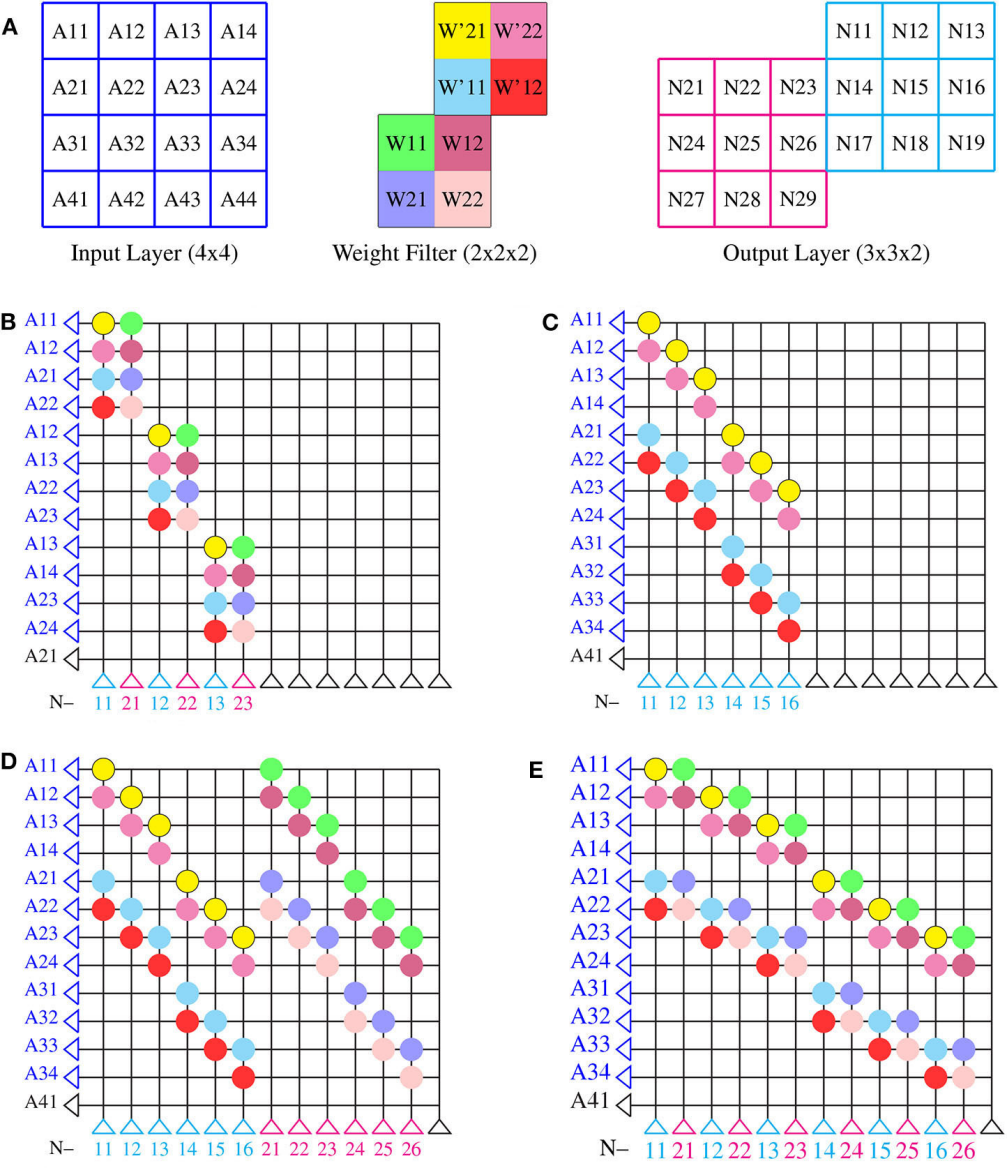
Methods of mapping onto a single neuromorphic core: **(A)** an example of convolution operation between layers for illustrating different mapping techniques. Input of size 4 × 4 and filter weights of size 2 × 2 × 2 is considered for convolution to obtain an output of size 3 × 3 × 2. **(B)** Block method, **(C)** toeplitz method and hybrid method, **(D)** toeplitz method in blockwise manner, and **(E)** block method in toeplitz manner.

Crossbar architecture is efficient for implementing a fully connected neural network and its mapping is straightforward. However, mapping a convolution layer in a CNN onto a crossbar architecture is not and requires further consideration for efficient mapping. An example of convolution operation in between layers of a CNN is illustrated in Figure 4A. We consider a 4 × 4 input layer. Convolution of this input layer with two weight filters of size 2 × 2 and stride of 1 will result in an output layer of size 3 × 3 × 2. If this same network was considered fully connected between input layer of 16 neurons and output layer of 18 neurons, then, for such a network of full connection, a core size of 16 × 18 is required, which will have 100% synaptic utilization in the core. The mapping of CNNs onto a crossbar array will, however, require a layer-wise core size of 4 × 18 in the above example, while the input will have to be fed in over many time steps, in order to have 100% synaptic utilization (with duplication of synaptic weights). Hence, CNN mapping onto crossbar architecture will always lead to some weights not being used and we want to explore what mapping techniques can reduce this wastage. Mapping convolution onto a crossbar architecture can be constructed by any of the methods as shown in Figure 4. Note the numbering of neurons in each layer along with the color of each weight for better illustration of the different methods of mapping.

- Block method: as shown in Figure 4B, one could see that mapping is done in a straightforward manner without optimization. Here, the input sequence is repeated in the rows of a crossbar array; the neurons across the feature maps in a layer are arranged in the columns of the crossbar array and the weights are laid down according to the connections of the input axons and output neurons. This kind of implementation results in the weight matrices being mapped onto the crossbar array in blockwise manner, hence the name. Each block of weight matrix in Figure 4B are repeated from the same layers with the weight matrix being flattened into a row with size of kernel width × kernel height × number of feature maps (2 × 2 × 2) in the layer. Throughout the crossbar array these weight blocks (2 × 2 × 2) are repeated diagonally. In this method, one can find that the neurons across feature maps (N11 and N21 are the first and second neurons, respectively) are selected for mapping in the crossbar array and hence early layers of convolution which contain less feature maps maybe implemented using this method for better hardware utilization.
- Toeplitz method: the weight matrices are arranged in the toeplitz matrix or circular matrix format as shown in Figure 4C. This optimized method of mapping is commonly used in a neuromophic core with crossbar array of synapses. Here, the neurons are selected from a single feature map of a particular layer instead of selecting neurons across feature maps (note that the neurons are chosen from first channel of output layer in cyan color). The corresponding axons are selected from the previous layer and is arranged sequentially without any repetitions. The weight matrix per column of a crossbar array is the flattened structure of weight matrix in a particular layer of a neural network architecture. This weight matrix per column repeats along each columns in a circular shift with respect to the strides of convolution in the particular layer as shown in Figure 4C. This method maps each feature map of a layer in crossbar arrays rather than mapping neurons across the feature maps as in block method. The toeplitz method of mapping is therefore suitable for certain type of convolutional layers, such as the depthwise convolutional layer, in which convolutions are separately performed in each feature map, independent of other feature maps within a layer (Howard et al., 2017; Gopalakrishnan et al., 2019a) or suitable for layer wise computations, such as pooling.
- Hybrid method: Considering the two aforementioned methods, one may combine the block and toeplitz methods of mapping in two different ways, as shown in Figures 4D,E. In Figure 4D, we select the neurons within a feature map (N11, N12, N13, etc. in cyan color from the same feature map of output layer) and lay down the weights in the toeplitz matrix manner. This toeplitz method is then repeated in a blockwise manner throughout the crossbar array, mapping a set of neurons across the feature maps of a particular layer in the neural network architecture. This can be viewed as implementing the toeplitz method in a blockwise manner. We can also implement block method in the toeplitz manner as shown in Figure 4E, where the neurons across a feature map (N11 in cyan and N21 in magenta as group of neurons from different feature maps of output layer) are selected for mapping using the block method, though without any repeated axons, while the entire block is repeated in a toeplitz manner throughout the crossbar array, mapping a set of neurons across the feature maps of a convolutional layer.

### 2.2. Co-design

#### 2.2.1. Core Matrix Splitting

For the toeplitz and hybrid method of mapping techniques, as shown in Figure 4, the axons of the neuromorphic core are arranged sequentially without any repetition as compared to the block method of mapping. Such sequential input of axons is possible only with the circular or toeplitz arrangement of weight filters in a crossbar architecture. In fact, the mapping of CNN layers onto crossbar architecture involves conversion of two-dimensional arrays into one-dimensional arrays. The two-dimensional convolutional operations in a CNN is converted to a one-dimensional convolutional operation along each columns of a crossbar architecture. The neurons in the output layer is mapped onto the crossbar array with its corresponding one-dimensional array of input axons in a sequence and extending the one-dimensional weight matrix along each column of crossbar by circularly shifting the one-dimensional weight matrix with respect to corresponding convolutional strides (as shown in Figure 4C). Two adjacent convolutional operation shares a portion of input section with respect to the strides. This shared portion of input while convolution is reflected as weight connections along the rows of the core. The number of weight connections along each row implies the fan-out of that particular axon in the core, whereas the weight connections along the column implies the fan-in of that particular neuron in the core. For the toeplitz method of mapping, a section of any CNN layer with a rectangular dimension of *neuron*_*row*_ and *neuron*_*col*_ to a crossbar array, the number of axons to be selected for such a section of CNN layer can be expressed mathematically:

(1) $$\begin{array}{r} N_{axons}=K\times K+K\times S\times (Neuron_{col}-1)+S\times S\times \\ (Neuron_{col}-1)\times (Neuron_{row}-1)+\\ K\times S\times (Neuron_{row}-1) \end{array}$$

where,

*N*_*axons*_ = total number of axons to be selected from layer N-1.

K = convolution filter size. Here, we have only considered same width and height for kernel filter size.

S = stride

*Neuron*_*row*_ = number of neurons across row to be selected from layer N.

*Neuron*_*col*_ = number of neurons across column to be selected from layer N.

To decide on core size, a major restriction comes from the below inequality:

(2) $$N_{axons}<Axon\;size,AS$$

where Axon size and AS the number of physical axons per core. In general, we would like to minimize AS to have smaller cores, and we thus need to minimize *N*_*axons*_. In this case, the selection of neurons, *Neuron*_*row*_ and *Neuron*_*col*_, in a layer becomes primary step in mapping. To perform the optimization, we further consider that output size (*O*_*size*_) is constant, i.e.,

(3) $$O_{size}=Neuron_{row}\times Neuron_{col}=A$$

where A ∈ R is a constant. The optimization problem can be now framed as choosing *Neuron*_*row*_ and *Neuron*_*col*_ to minimize Equation (1) subject to the constraint in Equation (2). Do note that Equation (1) considers only a single feature map; this can be easily extended to multiple feature maps by multiplying right hand side of Equation (1) with the respective number of feature maps in each layer.

Now, denoting *Neuron*_*row*_ and *Neuron*_*col*_ by x and y, respectively, for brevity of notation, Equation (1) can be reduced as follows:

(4) $$\begin{array}{r} N_{axons}=K\times K+K\times S\times (Neuron_{col}-1)+S\times S\times \\ (Neuron_{col}-1)\times (Neuron_{row}-1)+\\ K\times S\times (Neuron_{row}-1)\\ =K\times K+K\times S\times (y-1)+S\times S\times (y-1)\times (x-1)\\ +K\times S\times (x-1)\\ =K^{2}+KS(x+y-2)+S^{2}(xy-y-x+1)\\ =(K^{2}-2KS+AS^{2}+S^{2})+(x+y)(KS-S^{2}) \end{array}$$

where we have used xy = A from Equation (3). Since (KS −*S*^2^) > 0, minimizing Equation (4) is equivalent to minimizing (x+y). We show in the following theorem that (x+y) is minimized for x = y.

*Theorem*: For any given a ∈ R, then x = y, for argmin (x+y), such that x × y = A, x,y ∈ R.

*Proof* :

(5) $$\begin{array}{c} Let\;x+y=Z\\ then\;x+\frac{A}{x}=Z\\ \frac{dZ}{dx}=1-\frac{A}{x^{2}}\\ at\;minima\frac{dZ}{dx}=0,\\ \;∴1-\frac{A}{x^{2}}=0\\ x=\pm \sqrt{A}\\ If\;x,y>0,\;then\;Z\;is\;minimum\;∴x=y=\sqrt{A} \end{array}$$

From the theorem and its proof, we can see that for optimized mapping, a square shaped selection of neurons is always better. This implies that, for optimized mapping, *Neuron*_*row*_ = *Neuron*_*col*_ in Equation (1). Hence, substituting *Neuron*_*row*_ = *Neuron*_*col*_ = “*N*_*neurons*_” in Equation (1), we get,

(6) $$N_{neurons}=(\sqrt{N_{axons}}-K)/S+1$$

If we consider input channels in Equation (1), then the above equation becomes

(7) $$N_{neurons}=(\sqrt{N_{axons}}/C-K)/S+1$$

where, C = number of input channels for each layer.

This equation can be used to determine the design co-mapping of the convolutional neural network onto a neuromorphic chip with crossbar array of synapses, where the hardware constraint is given by the axon size and the convolution layer design is with respect to K, S, and C for each layer. This suggests that mapping and designing of a CNN must co-exist for the better utilization of a neuromorphic hardware.

It can also be seen from the optimized mapping that the time delay of the hardware design is less. The design space exploration of core size, [axon size (AS) × neuron size (NS)] w.r.t *N*_*axons*_, *Neuron*_*row*_, and *Neuron*_*col*_ becomes [*N*_*axons*_×(*Neuron*_*row*_×*Neuron*_*col*_)] = [$N_{axons}\times N_{neurons}^{2}$]. This implies that the search space for neuron size is reduced to only square numbers ($N_{neurons}^{2}$) instead of all the factors (*Neuron*_*row*_ × *Neuron*_*col*_) of the neurons in a core (NS = 256, 512, etc). The fact that a unique HFNet is trained for each topology saves on many days of training.

**Figure 5:**
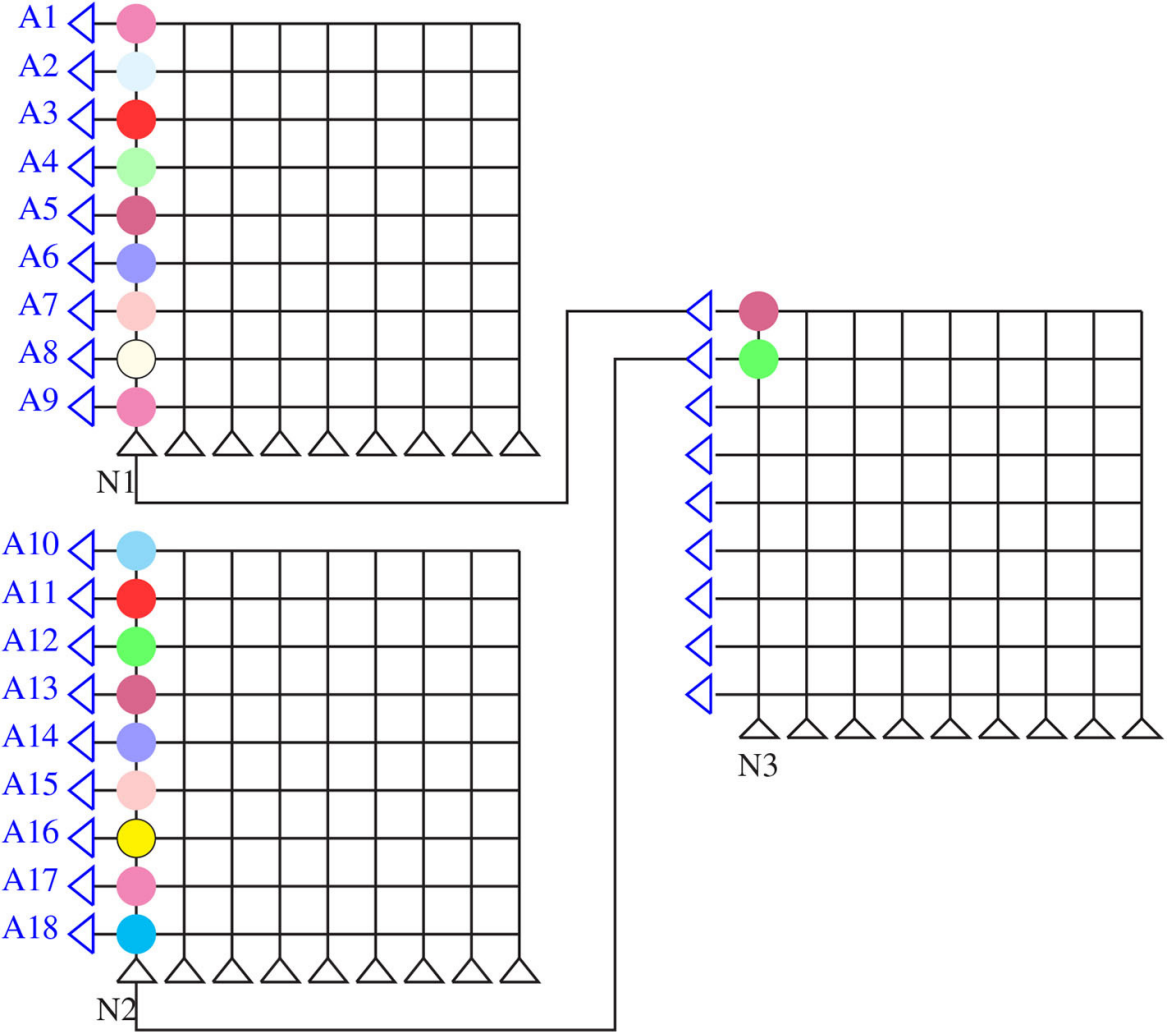
Core matrix splitting: splitting of weighted sum of inputs into two cores and summing up of intermediate activations in a third neuromorphic core.

In the event that the fan-in degree of a single neuron in a layer exceeds the maximum number of axons in a neuromorphic core [*N*_*axons*_ > AS in Equation (1) when *Neuron*_*row*_ = 1 and *Neuron*_*col*_ = 1], mapping of that particular neuron has to be split among multiple cores, as shown in Figure 5. In general, if the output of each neuron undergoes a non-linear activation, the final output would deviate from the intended output:

(8) $$\begin{array}{l} given,\;W=(W_{1},W_{2}),\\ f(f(\sum W_{1}A)+f(\sum W_{2}A)\neq f(\sum WA)) \end{array}$$

This method of splitting is termed as core matrix splitting (Figure 5). Additional hardware considerations have to be made to communicate only intermediate results without the activation function. Additional cores are also required for mapping. In order to avoid core matrix splitting, a hardware-friendly approach is considered by grouping neurons across feature maps while training in the IBM TrueNorth chip (Esser et al., 2016). In this work, we adopt a different approach by modifying existing CNN architectures and training them. We consider other forms of convolution operations, such as depthwise and pointwise convolutions [network-in-network (Lin et al., 2013) and MobileNet (Howard et al., 2017)] while co-designing the CNN with the core size in mind so as to avoid core matrix splitting.

#### 2.2.2. Overview of Convolution Layers in a CNN

Standard convolution can be computationally intensive and also hard to map onto square neuromorphic cores of limited sizes. To map them, we would have to consider cores of different shapes. Given these square cores, we considered other computationally less intensive convolution techniques, namely, depthwise, pointwise, and group convolution.

##### 2.2.2.1. Depthwise convolution

In depthwise convolution, the convolution operation is independently applied to each input channel so as to obtain its corresponding output feature map (Howard et al., 2017). In general, the number of output channels from a depthwise convolution is the same as the number of input channels, although this may be changed by outputting multiple channels per input channel (depth multiplier parameter). The depthwise convolution is typically followed by pointwise convolution, which is discussed in section 2.2.3. For depthwise convolution, weights from within rather across feature maps are mapped first. Hence, the toeplitz method is better suited for mapping depthwise convolutions.

**Figure 6:**
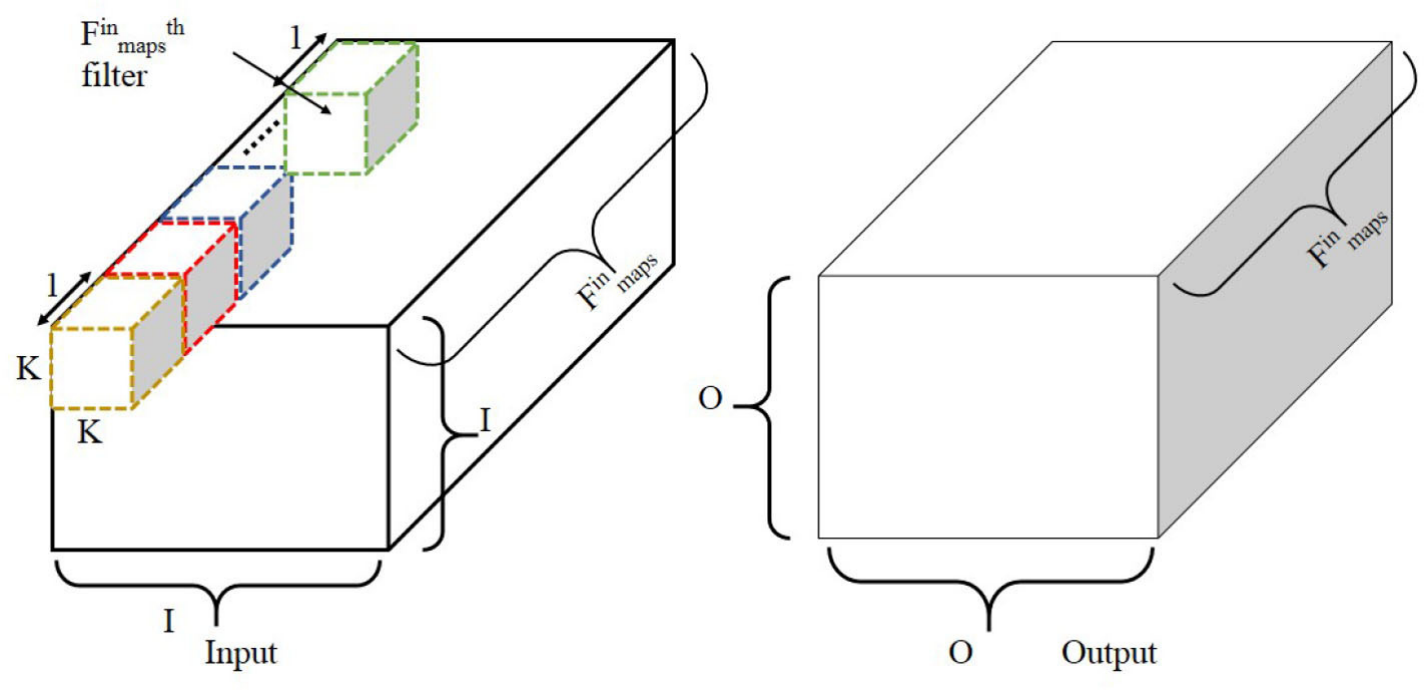
Illustration of depthwise convolution. Note that depth multiplier is set to one here. The filter size is K × K × 1 with $F_{maps}^{in}$ such filters to obtain an output size of O × O$\times F_{maps}^{in}$. Adapted from Gopalakrishnan et al. (2019a).

As shown in Figure 6, the input matrix is convolved with $F_{maps}^{in}$ different filters, each of size K × K. The output of each depthwise convolution involving a filter and a single input channel is O × O × 1, and $F_{maps}^{in}$ such filters compute an output of dimensions O × O × $F_{maps}^{in}$. The depth multiplier is set to one here. The computational cost, C, of depthwise convolution is given below:

(9) $$C=O^{2}\times K^{2}\times F_{maps}^{in}\times D$$

where,

O = output size after convolution

K = filter size

$F_{maps}^{in}$ = number of input channels

D = depth multiplier.

##### 2.2.2.2. Pointwise convolution

Pointwise convolution is a special case of the standard convolution operation whereby the filter size per channel is 1 × 1 (Lin et al., 2013). The entire filter therefore has a shape of 1 × 1 × $F_{maps}^{in}$ × $F_{maps}^{out}$, where $F_{maps}^{in}$ is the number of input channels and $F_{maps}^{out}$, the number of output channels. Since the filter size is reduced, the computational complexity is also reduced by an order of the square of the filter size. Its computational cost, C, is given below:

(10) $$C=O^{2}\times F_{maps}^{in}\times F_{maps}^{out}$$

where,

O = output size after convolution

$F_{maps}^{in}$ = number of input channels

$F_{maps}^{out}$ = number of output channels.

While we can use either the toeplitz or hybrid method of mapping for pointwise convolution layers depending on the CNN architecture and the core sizes, the block method in toeplitz is preferred if AS < NS; otherwise the toeplitz method in block is preferred (AS > NS).

##### 2.2.2.3. Grouped convolution

**Figure 7:**
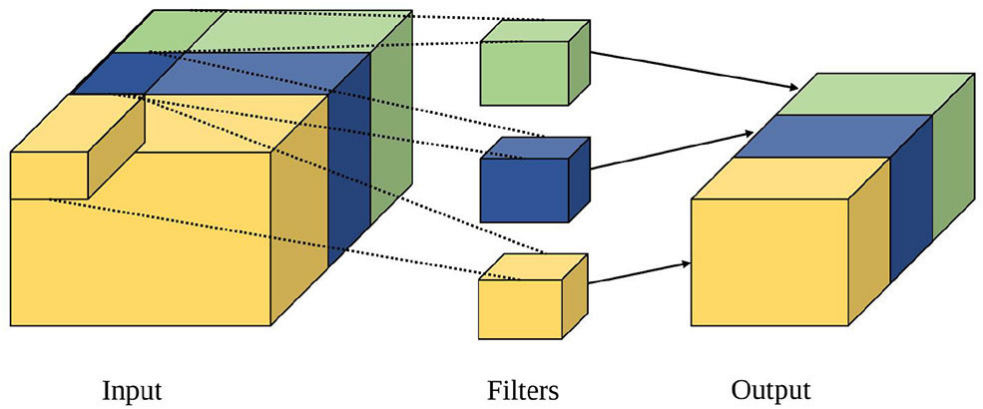
Illustration of group convolution adapted from Gopalakrishnan et al. (2019a).

Grouped convolution is a convolution technique whereby the standard convolution is applied separately to an input matrix diced into equal parts along the channel axis. As shown in Figure 7, the input is divided into equal parts along the channel axis, and group convolution is then applied separately. The individual outputs are then combined into a final output, with variations, such as stacked convolution, dependent stacked convolution and shuffled group convolution (Zhang and Sun, 2018). Computational complexity of grouped convolution is calculated as per standard convolution. It is therefore more hardware friendly as each neuron has a lower fan-in/fan-out degree when mapped. Either the toeplitz or hybrid method of mapping may be used for grouped convolutions.

#### 2.2.3. Insights From Different CNNs

##### 2.2.3.1. MobileNet

Depthwise and pointwise convolutions and depthwise separable convolutions are introduced in MobileNet (Howard et al., 2017). For pointwise convolution, one may think of it as duplicates of full connections between inputs and outputs in the same location channel-wise. This is ideal for efficient mapping onto crossbar array of synapses with good core utilization. The application of depthwise and then pointwise convolution (depthwise separable convolution) has a much lower fan-in degree per neuron compared to the standard convolution, which helps to avoid core matrix splitting. As such, depthwise separable convolution is the preferred method in our co-design of CNN architectures.

##### 2.2.3.2. VGGNet and NIN

VGGNet (Simonyan and Zisserman, 2014) gradually shrinks the size of feature maps by applying max pooling after two convolutions in the shallow layers and every three layers afterwards. Intuitively, this approach improves classification accuracy, which we also validate as shown in results in section 3. Using fewer feature maps when each map is large effectively reduces the fan-in degree of the neurons and avoid core matrix splitting.

##### 2.2.3.3. Other insights

Global average pooling (GAP) as used in Network in network (NIN) (Lin et al., 2013) or SqueezeNet (Iandola et al., 2016) helps to reduce fan-in degree of neurons. Instead of using fully connected layers in the deeper layers of the CNN, which have high fan-in degree, one may use GAP for a more hardware-friendly design. Maxpooling is also not hardware friendly. The toeplitz mapping method is required for maxpooling, resulting in poor core utilization. We would therefore avoid maxpooling when co-designing the hardware-friendly CNN. The reduction in feature map size achieved by maxpooling may also be achieved by increasing the stride size of prior convolution layer with no significant loss in accuracy, even if functionally, they are different (Springenberg et al., 2014). Hence, the above insights (listed below) help to guide our co-design of the hardware-friendly CNN (section 2.2):

- Combined usage of standard convolution and depthwise separable convolutions,
- Excluding pooling between standard convolutions,
- And replacement of fully connected layers with GAP at end of CNN.

#### 2.2.4. Co-design Methodology

##### 2.2.4.1. Hardware-friendly CNN

**Figure 8:**
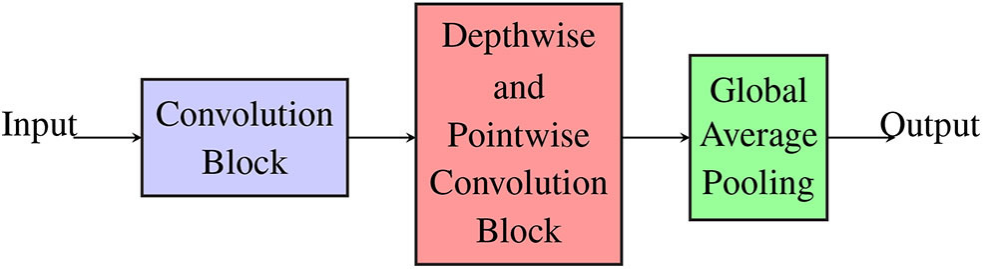
Hardware-friendly CNN in modular form: standard convolution, depthwise separable convolution, and global average pooling.

**Figure 9:**
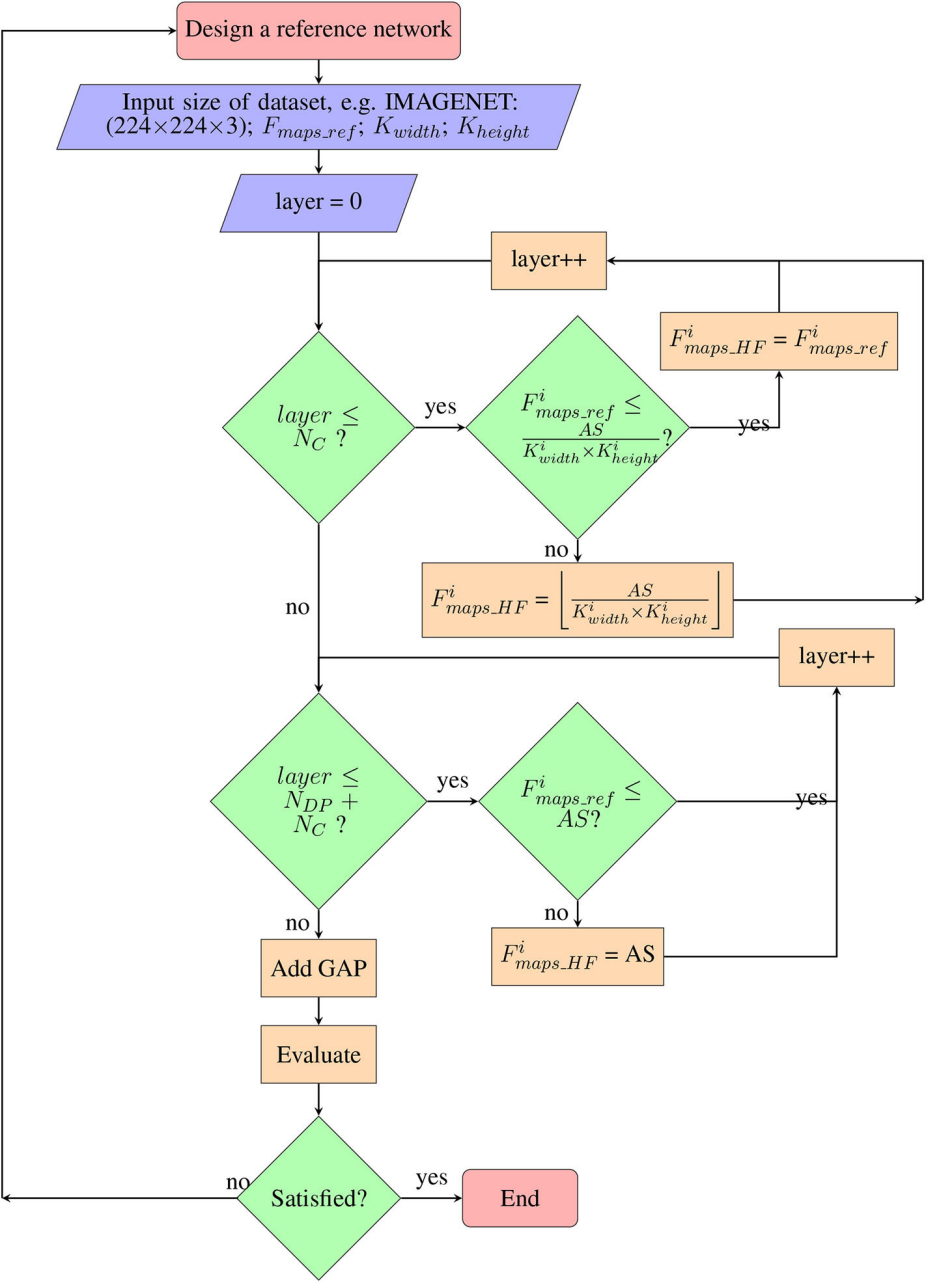
Co-design flowchart: step by step methodology for co-design of hardware-friendly CNN.

We adopt the modular form of the CNN as shown in Figure 8 for a hardware-friendly architecture. The iterative process of the co-design is described in Figure 9. We first initiate certain design parameters, such as “*N*_*C*_” number of convolutional layers and “*N*_*DP*_” number of depthwise separable convolution layers. Setting *N*_*C*_ and *N*_*DP*_ depends on the input size and the stride size of each convolution, which affects the classification accuracy of the CNN. The singular GAP layer is added to end of the CNN.

The complete step by step co-design process is shown in the flowchart (Figure 9):

- After setting the number of layers in each module, we next decide on the number of feature maps per layer, with the constraint of avoiding core matrix splitting (Figure 9). $F_{maps\text{\_}HF}^{i}$ (HF denotes hardware friendly) for each layer i depends on the number of axons in each core (axon size, AS) and constraining the fan-in degree of a neuron in i (fan-in ≤ AS) to avoid core matrix splitting. For the standard convolution layer, the number of feature maps denoted by *F*_*maps*_ can be calculated as below:
  (11) $$F_{maps}\leq \frac{AS}{K_{width}\times K_{height}}$$
  where,
  *K*_*width*_ and *K*_*height*_ are respectively the width and height of the convolution kernel.
  AS = axon size of crossbar array in a neuromorphic core.
  Equation (11) is the maximum possible number of feature maps, *F*_*maps*_, when in Equation (1) we set *Neuron*_*row*_ = 1 and *Neuron*_*col*_ = 1. By so doing, we set the reference number of feature maps in i, $F_{maps\text{\_}ref}^{i}$ to be less than the fan-in (maximum possible axon connections) degree of a neuron. In such a case, toeplitz mapping may also be used.
- For pointwise convolution, we need to ensure that *F*_*maps*_ ≤ axon size. Core matrix splitting is not an issue for depthwise convolutions with small kernel size. Hence, for depthwise separable layers, fan-in degree of pointwise convolution is the key delimiting factor. The co-designed CNN is then mapped to obtain number of cores used, and it is then trained and tested for classification accuracy (section 2.3). If it is not satisfactory, the process is repeated with different initial parameters, *N*_*C*_ and *N*_*DP*_.

### 2.3. CNN Training and Mapping

#### 2.3.1. CNN Training

The co-designed CNNs are trained using Tensorpack (Wu, 2016). We use “Momentum” Optimizer (momentum of 0.9) with batch size of 48, and weight regularization with decay of 0.0005. We initialized the weights using “He normal” initializer. The learning rate is adjusted using a heuristic whereby it is reduced by 10 every 30 epochs. The learning rate was initialized at 0.01 and reduced three times prior to termination. We trained the network for 100 epochs on the IMAGENET dataset. Accuracy is chosen from the best testing accuracy across five trials.

#### 2.3.2. CNN Mapping

The calculation for the number of cores is obtained using the python wrapper, mapping, and debugging (MaD) framework (Gopalakrishnan et al., 2019b), which also map the CNN onto cores. The mapping function outputs the weight matrices for the crossbar array in each core, a connectivity list between cores and an estimate of total number of cores needed for mapping. MaD also allows us to carry out inference in Python on the core level, which is useful for validating the correctness of the mapping done, and also study communication across cores, such as traffic volume and energy consumption estimation.

The methods and techniques mentioned in this paper is not restricted to any kind of neural network like ANN or SNN. The mapping and designing methods are useful for both ANN and SNN, especially with convolutional layers in their architecture. In fact, the simulations are all done for ANN models and not SNN. All the codes used for generating results in this manuscript is publicly available at https://github.com/roshan-gopalakrishnan/NeuromorphicComputing.git.

## 3. Experimental Framework and Results

This section is also divided into two subsections: mapping and a proposed CNN, the HFNet. In section 3.1, we benchmark the different mapping techniques based on cores used. In 3.2, we propose a hardware-friendly CNN, the HFNet, and report on (1) the cores required for mapping, (2) classification accuracy and cores required with and without maxpooling and full connections, (3) classification accuracy and cores required for different core sizes, (4) comparison of the MobileNet and HFNet, and (5) the results when grouped convolution replaces depthwise separable convolution.

### 3.1. Mapping Results

**Figure 10:**
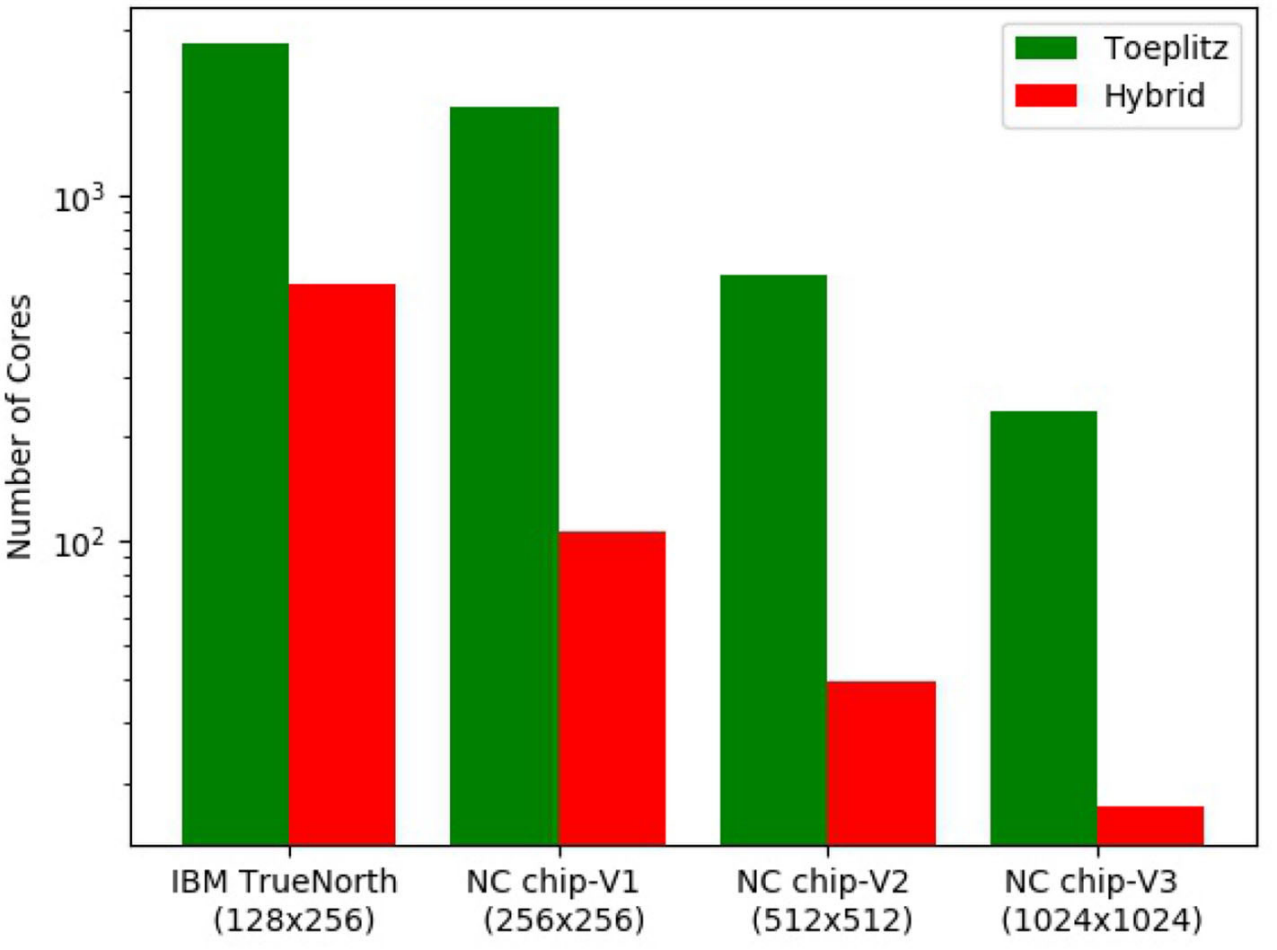
Benchmarking of mapping methods. The bar graph shows the number of cores utilized for both hybrid and toeplitz mapping against different core sizes. We consider only the convolution layers in the CNN.

We first study the advantage of using the hybrid method over the toeplitz method of mapping a CNN for classifying CIFAR-10 (Krizhevsky, 2009) (Figure 10). As mapping of pooling layers is done with the toeplitz method and mapping fully connected layer requires core matrix splitting, we consider only the convolutional layers for illustrative purposes. The CNN is designed in the following manner: 32 × 32 × 3 (stride 2) – 16 × 16 × 4 – 14 × 14 × 8 – 12 × 12 × 12 – 10 × 10 × 16 – 8 × 8 × 20 – 6 × 6 × 24 – 4 × 4 × 28. All 7 convolutional layers have kernel filter size 3 × 3 and stride of 1, unless otherwise stated. We consider four different core sizes for mapping: 128 × 256 (similar to IBM TrueNorth), 256 × 256 (NC chip-V1), 512 × 512 (NC chip-V2), and 1,024 × 1,024 (NC chip-V3). The benchmarking metric is the number of cores needed to map the CNN. From the bar graph in Figure 10, it is observed that hybrid method always utilize less number of cores compared to the toeplitz method and number of cores required decrease with core sizes, irrespective of mapping methods. Quantitatively, 13.88, 15, 16.98, and 4.94 times fewer cores are utilized in the case of hybrid mapping compared to toeplitz mapping on neuromorphic chip with core sizes 128 × 256, 256 × 256, 512 × 512, and 1,024 × 1,024, respectively.

### 3.2. Hardware-Friendly CNN: The HFNet

Using the co-design process as described in section 2.2, we propose the HFNet. It is a hybrid architecture, based on our insights (Figure 8), that borrows from the VGGNet, MobileNet, and NIN so as to improve mapping on neuromorphic cores. As shown in Figure 8, the shallow layers are standard convolutional layers (VGGNet), followed by depthwise separable convolutions (MobileNet), and fully connected layers with large fan-in degrees are replaced with GAP (NIN).

The detailed input, output size of each layer in the HFNets for different core sizes are given in Table 1. We have named different versions of the HFNets for different core sizes (256 × 256, 512 × 512, and 1,024 × 1,024, respectively) as HFNet-V1, HFNet-V2, and HFNet-V3. Core size determines the number of axons (AS) × number of neurons (NS) in a single core. The second convolution layer in HFNet-V2 (1) has *F*_*maps*_ = 56, which is the maximum it can have (Equation 11) to avoid core matrix splitting. A kernel size of 3 × 3 is used for standard and depthwise convolution.

**Table 1:** Neural network architecture (NN archi.) for different core sizes.

**NN**	**HFNet model (core size)**					
**archi**.	**HFNet-V1 (256** **× ** **256)**		**HFNet-V2 (512** **× ** **512)**		**HFNet-V3 (1,024** **× ** **1,024)**	
**Layers**	**Input size**	**Output size**	**Input size**	**Output size**	**Input size**	**Output size**
C^a^	226 × 226 × 3	112 × 112 × 16	226 × 226 × 3	112 × 112 × 32	226 × 226 × 3	112 × 112 × 32
C	114 × 114 × 16	56 × 56 × 28	114 × 114 × 32	56 × 56 × 56	114 × 114 × 32	56 × 56 × 64
C	58 × 58 × 28	28 × 28 × 64	58 × 58 × 56	28 × 28 × 256	58 × 58 × 64	28 × 28 × 256
D^b^	30 × 30 × 64	28 × 28 × 64	30 × 30 × 256	28 × 28 × 256	30 × 30 × 256	28 × 28 × 256
P^c^	28 × 28 × 64	28 × 28 × 256	28 × 28 × 256	28 × 28 × 256	28 × 28 × 256	28 × 28 × 256
D	30 × 30 × 256	14 × 14 × 256	30 × 30 × 256	14 × 14 × 256	30 × 30 × 256	14 × 14 × 256
P	14 × 14 × 256	14 × 14 × 256	14 × 14 × 256	14 × 14 × 512	14 × 14 × 256	14 × 14 × 512
D	16 × 16 × 256	14 × 14 × 256	16 × 16 × 512	14 × 14 × 512	16 × 16 × 512	14 × 14 × 512
P	14 × 14 × 256	14 × 14 × 256	14 × 14 × 512	14 × 14 × 512	14 × 14 × 512	14 × 14 × 512
D	16 × 16 × 256	14 × 14 × 256	16 × 16 × 512	14 × 14 × 512	16 × 16 × 512	14 × 14 × 512
P	14 × 14 × 256	14 × 14 × 256	14 × 14 × 512	14 × 14 × 512	14 × 14 × 512	14 × 14 × 1,024
D	16 × 16 × 256	14 × 14 × 256	16 × 16 × 512	14 × 14 × 512	16 × 16 × 1,024	14 × 14 × 1,024
P	14 × 14 × 256	14 × 14 × 256	14 × 14 × 512	14 × 14 × 512	14 × 14 × 1,024	14 × 14 × 1,024
D	16 × 16 × 256	14 × 14 × 256	16 × 16 × 512	14 × 14 × 512	16 × 16 × 1,024	14 × 14 × 1,024
P	14 × 14 × 256	14 × 14 × 256	14 × 14 × 512	14 × 14 × 512	14 × 14 × 1,024	14 × 14 × 1,024
D	16 × 16 × 256	14 × 14 × 256	16 × 16 × 512	14 × 14 × 512	16 × 16 × 1,024	14 × 14 × 1,024
P	14 × 14 × 256	14 × 14 × 256	14 × 14 × 512	14 × 14 × 512	14 × 14 × 1,024	14 × 14 × 1,024
D	16 × 16 × 256	7 × 7 × 256	16 × 16 × 512	7 × 7 × 512	16 × 16 × 1,024	7 × 7 × 1,024
P	7 × 7 × 256	7 × 7 × 1,000	7 × 7 × 512	7 × 7 × 1,000	7 × 7 × 1,024	7 × 7 × 1,024
D	9 × 9 × 1,000	7 × 7 × 1,000	9 × 9 × 1,000	7 × 7 × 1,000	9 × 9 × 1,024	7 × 7 × 1,024
P	7 × 7 × 1,000	7 × 7 × 1,000	7 × 7 × 1,000	7 × 7 × 1,000	7 × 7 × 1,024	7 × 7 × 1,000
GAP^d^	7 × 7 × 1,000	1 × 1 × 1,000	7 × 7 × 1,000	1 × 1 × 1,000	7 × 7 × 1,000	1 × 1 × 1,000

a *Convolution layer*.

b *Depthwise convolution layer*.

c *Pointwise convolution layer*.

d *Global average pooling*.

#### 3.2.1. Maxpooling and Fully Connected Layers

This experiment investigates the performance of the proposed architecture with and without maxpooling and fully connected layers (Table 1). HFNet-V3 with maxpooling replaces all convolution layers with stride of two with convolution layer with stride of 1 and an additional maxpooling layer. For HFNet-V3 with full connections, a fully connected layer (1,024 × 1,000) is added on top of the average pooling layer in HFNet-V3 while changing the last pointwise convolution layer to 7 × 7 × 1,024 instead of 7 × 7 × 1,000. From Table 2, it can be seen that there is only very slight improvement in classification accuracies when maxpooling or full connection is used. This further validates our design criteria for HFNet. The number of cores required for HFNet-V3 with pooling layer is huge, as mapping of pooling layers is done using the toeplitz method. The number of parameters is higher for HFNet-V3 with fully connected layer. All CNNs considered in this experiment is illustrated in Supplementary Material.

**Table 2:** With and without pooling and fully connected layers.

	**HFNet-V3**	**With pooling layer**	With FC^*^ layer
Classification accuracy (%)	71.3	71.6	71.5
Number of parameters (M)	6.46	6.46	7.51
Storage for parameters (MB)	24.63	24.63	28.63
Number of cores (1,024 × 1,024)	4,720	10,940	4,721

* *Fully connected*.

#### 3.2.2. Number of Cores and Classification Accuracy

**Figure 11:**
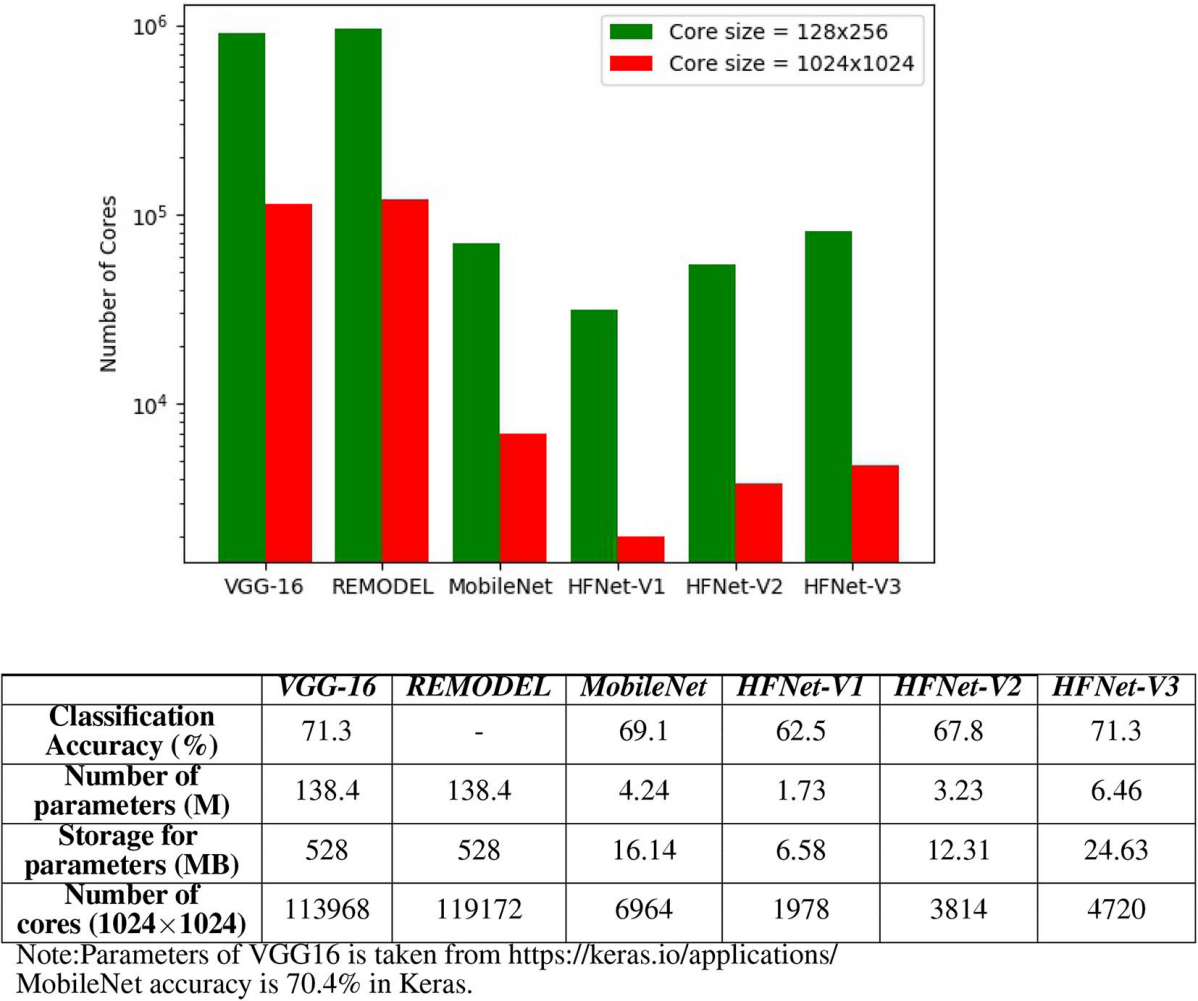
Comparing different CNNs: the bar graph shows the number of cores required for mapping different CNNs. The table shows the classification accuracy on IMAGENET for the CNNs. Classification accuracy of HFNet-V3 is close to VGG-16 and MobileNet, while requiring a smaller number of cores.

Here we study both the number of cores required for mapping and the classification accuracy (IMAGENET) for the HFNets (Figure 11) and three other popular CNN architectures, namely VGGNet (VGG-16), MobileNet, and REMODEL [a modification of VGG-16 for mapping the final fully connected layers onto IBM TrueNorth (Shukla et al., 2019)]. We consider two core sizes: the minimum 128 × 256 and the maximum size 1,024 × 1,024. Note that VGG-16 and REMODEL require core matrix splitting for mapping onto a 1,024 × 1,024 core. Mapping of all CNNs onto 128 × 256 cores requires core matrix splitting. As expected, the number of cores used by MobileNet and HFNet are ~10 times fewer compared to VGG-16 and REMODEL. It can be seen that HFNet-V1 uses the least number of cores among all models. The table (Figure 11) shows the classification accuracy of the CNNs on IMAGENET. The HFNet-V3 is as accurate as VGG-16 while 2.2% more accurate than the MobileNet. It also utilizes less number of cores than the MobileNet. All HFNet models considered in this experiment is illustrated in Supplementary Material.

#### 3.2.3. Augmenting the HFNet

**Figure 12:**
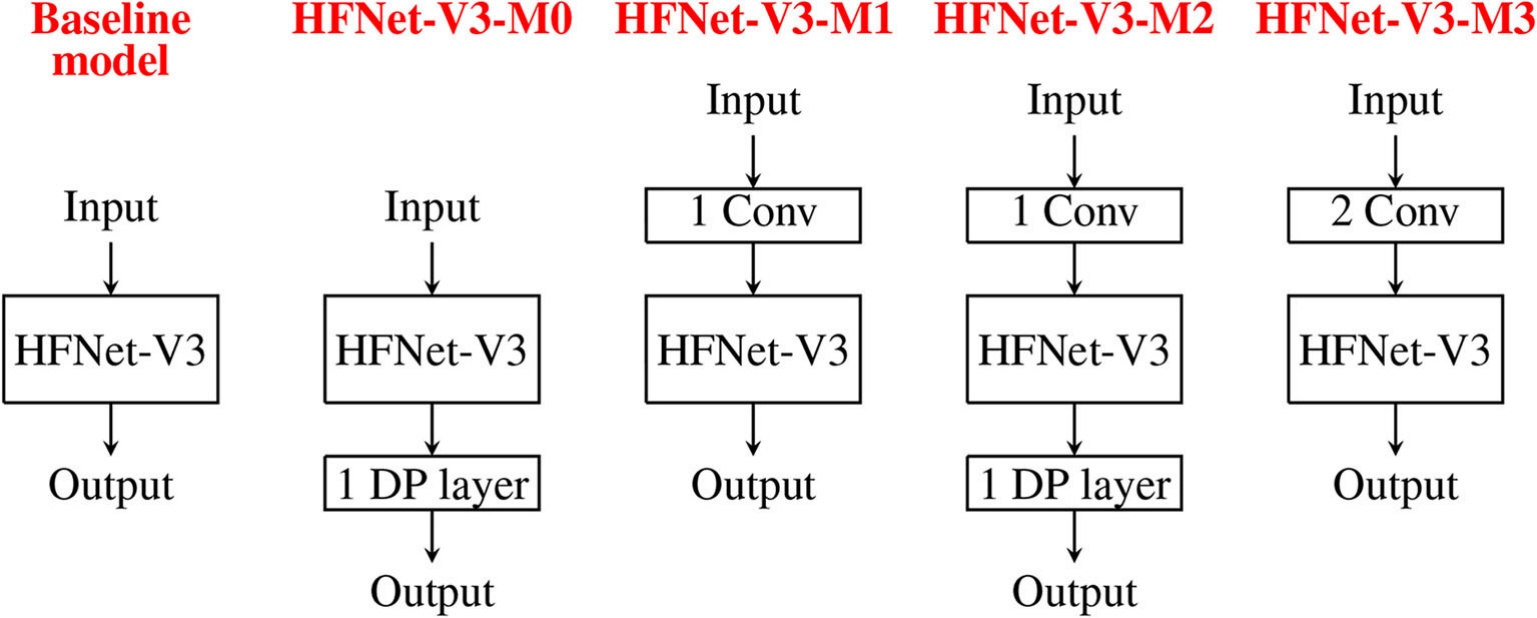
Illustration of adding different convolutions to the baseline model, HFNet-V3: HFNet-V3-M0 has one more depthwise separable convolution. HFNet-V3-M1 has one more standard convolution. HFNet-V3-M2 has one more standard and one more depthwise separable convolution. HFNet-V3-M3 has two more standard convolutions.

This experiment adds more hardware-friendly layers to HFNet-V3 to investigate the increase in classification accuracies. We report the accuracy results for the adding depthwise separable convolution layer to end of HFNet-V3 (HFNet-V3-M0), one or two standard convolution layers to front of HFNet-V3 (HFNet-V3-M1 and HFNet-V3-M3, respectively) and both these two layers (HFNet-V3-M2). Figure 12 illustrates these additions. Table 3 shows the corresponding results. As expected, the additional layers lead to improved accuracies, with a standard convolution layer having a larger impact than depthwise separable convolution, while having less total number of parameters. This further validates the insight from VGGNet: the retaining of larger feature maps at shallow layers improves accuracy. It can be seen that adding depthwise separable convolution layers has a larger increase in number of parameters compared to standard convolution in shallow layers but has a smaller increase in number of cores. Adding two standard convolutions does not lead to better accuracy compared to one standard and one depthwise separable convolution. Best accuracy is obtained for HFNet-V3-M2 among all variants of the HFNet. All CNNs considered are illustrated in Supplementary Material.

**Table 3:** Addition of layers to HFNet-V3.

	1 DP^*^ layer	**1 Conv layer**	**1 DP + 1 Conv layer**	**2 Conv layer**
	**HFNet-V3-M0**	**HFNet-V3-M1**	**HFNet-V3-M2**	**HFNet-V3-M3**
Classification	71.5	72.2	72.7	72.6
accuracy (%)				
Number of	7.52	6.64	7.7	6.75
parameters (M)				
Storage for	28.68	25.3	29.35	25.73
parameters (MB)				
Number of	4855	6960	6948	11664
cores (1,024 × 1,024)				

* *Depthwise separable convolution*.

#### 3.2.4. Comparison With Modified MobileNets

In this experiment, we compare the HFNets with modified MobileNets that are more hardware friendly. The MobilNet is chosen as it also uses the hardware-friendly depthwise separable convolution which also uses less parameters. The modified MobileNets are HF-MobileNet-V1 and HF-MobileNet-V2, such that the number of parameters are close to HFNet-V3-M1 and HFNet-V3, respectively. We also compare MobileNet with modified version of HFNet-V2, HFNet-V2-M0, by increasing parameters of HFNet-V2 to be close to those of MobileNet. From Table 4, the performance of HF-MobileNet-V2 (71.4%) compared to HFNet-V3 (71.3%) is better by only 1% while MobileNet (69.1%) compared to HFNet-V2-M0 (70%) is worse by 0.9% and HF-MobileNet-V1 (71.9%) compared to HFNet-V3-M1 (72.2%) is worse by 0.3%. Here, all the classification accuracies are trained using Tensorpack and Tensorflow framework. Note that MobileNet accuracy in Keras is 70.4%, 1.3% higher compared to Tensorpack result, but for a fair comparison we report accuracy results for CNN's trained using Tensorpack. Comparing MobileNets and HFNets with similar accuracy and parameter size, HFNet-V3 utilizes 2,809 less cores than MobileNet-V2, HFNet-V2-M0 utilizes 3,015 less cores than MobileNet and HFNet-V3-M1 utilizes 682 fewer cores than MobileNet-V1. The results show that the accuracies are close while there is a big difference in the number of cores utilized to make HFNet variants more hardware friendly. HFNets are therefore more neuromorphic hardware friendly than the hardware-friendly versions of existing deep learning architectures like MobileNet. The difference in number of cores for mapping is compared in Supplementary Material with respect to core utilization in each layer for HFNet-V3 and MobileNet.

**Table 4:** Comparison of HFNets with hardware-friendly MobileNets.

	**HFNet-**	**HF-**	**HFNet-V3**	**HF-**	**HFNet-**	**MobileNet**
	**V3-M1**	**MobileNet-V1**		**MobileNet-V2**	**V2-M0**	
Classification	72.2	71.9	71.3	71.4	70	69.1
accuracy (%)						
Number of	6.64	6.62	6.46	6.42	4.21	4.24
parameters (M)						
Storage for	25.3	25.22	24.63	24.46	16.05	16.14
parameters (MB)						
Number of	6,960	7,642	4,720	7,529	3,949	6,964
cores (1,024 × 1,024)						

*MobileNet accuracy is 70.4% in Keras*.

#### 3.2.5. Grouped Convolution

Here we replace depthwise separable convolution with grouped convolution (Esser et al., 2016): HFNet-GC. HFNet-GC is compared with HFNet-V3-M2, as it is modified from HFNet-V3-M2 by using approximately the same parameter size in each layer. To do so, we set the group convolutions to eight groups in each HFNet-GC layer. From Table 5, classification accuracy for HFNet-GC is around 13% less than the HFNet-V3-M2, while number of cores required almost doubled. HFNets with depthwise separable convolutions are hence more hardware friendly than HFNets with grouped convolutions. The architecture of HFNet-GC is illustrated in the Supplementary Material.

**Table 5:** Comparison of HFNet with grouped convolutions (GC).

	**HFNet-V3-M2**	**HFNet-GC**
Classification	72.7	59.8
accuracy (%)		
Number of	7.7	6.91
parameters (M)		
Storage for	29.35	26.38
parameters (MB)		
Number of	6,948	11,424
cores (1,024 × 1,024)		

## 4. Discussion and Conclusion

In our work, we first study what convolutions are more hardware friendly and how to best map them onto a neuromorphic hardware. We next identify deep learning techniques to avoid which result in poor core utilization or even result in core matrix splitting. We then propose a framework for the design of more hardware-friendly CNNs, and implement it using a Python wrapper (MaD). As a result of the above, the HFNet is proposed. Different versions of the HFNet are also proposed using the framework, that have better classification accuracy with more cores used when mapped. The framework thus allows us to propose different CNNs in a more principled manner by changing design parameters. We then evaluate it by comparison with other CNNs in terms of classification accuracy on IMAGENET, number of parameters, and cores required for mapping. It is able to achieve very comparable accuracy, using about the same number of parameters as per other more hardware-friendly CNNs but with substantially fewer mapped cores. Here, we have shown results on one of the biggest and most popular datasets in visual classification, IMAGENET, our results are quite generally applicable for visual tasks which is already covering a very big application space. Second, it has been shown that networks pre-trained on IMAGENET can be used as feature extractors for spectrograms for audio analysis (Acharya and Basu, 2020); our method can thus potentially generalize to other types of datasets as well. We have also proposed an optimized mapping technique by considering a square shaped selection of neurons. As shown in Table 6, we further explore how different core shapes (64 × 4,096, 128 × 2,048, 256 × 1,024, 512 × 512, 1,024 × 256, 2,048 × 128, 4,096 × 64) affect the number of cores used to map the HFNet-V3. Overall, the trend in cores used agree with the intuition provided in the proof (section 2.2.1), which is based on real numbers; while core sizes are based on integers which may lead to some discrepancy. We further note that cores required is not symmetric about 512 × 512, with larger input dimensions using less cores (e.g., 4,096 × 64 against 64 × 4,096). This is due to avoiding core matrix split while mapping.

**Table 6:** Number of cores for different core shapes.

**Core shape**	**Number of cores**
64 × 4,096	111,642
128 × 2,048	58,098
256 × 1,024	26,424
512 × 512	14,263
1,024 × 256	14,069
2,048 × 128	28,240
4,096 × 64	56,480

The HFNet is a hardware-friendly CNN that is designed using an iterative process that takes into account how best it can be mapped on a neuromorphic hardware with crossbar array of synapses while achieving good accuracy. One hard constraint while mapping is avoiding core matrix splitting. As shown in section 3, a typical HFNet requires thousands of cores for mapping. This is still larger than most neuromorphic chips. For future work, we will consider how one may map the HFNet onto a neuromorphic chip with limited number of cores. While we try to optimize synaptic resources and reduce “wastage” by minimizing unused synapses, it might be possible to reuse these synapses to provide a degree of fault tolerance in the hardware by providing redundancy. Current explorations of fault tolerance mostly show reduction in performance degradation after retraining a neural network with faults (Lee et al., 2014; Feinberg et al., 2018); however, there might be scope to optimize the fault tolerance by providing some extra synapses. We feel this is an important avenue of future work. We would also consider quantized CNNs, both weight and activations, in future work, which is beyond the scope of current work. Other hardware constraints, such as synaptic noise in novel devices, will be considered in future work.

Considering chip area, it maybe that only one physical neuron is implemented per core. This neuron is utilized in a time multiplexed manner to emulate all neurons within the core. If, however, there is more than one physical neuron per core, one can speed up computation by pipelining the time multiplexed neurons. Further speed-up can be achieved if the number of fan-in neurons per convolution operation is considered while increasing the physical neurons.

In our current study, we only studied CNNs without skip connections. Residual networks (He et al., 2016) have skip connections that increase the fan-in/fan-out degree of neurons. Not only that, to map them, we would either have to save intermediate results of skip-connections at routers or in buffers at the axons. This would be interesting to consider in future work.

## Data Availability Statement

All datasets generated for this study are included in the article/Supplementary Material.

## Author Contributions

RG and YC designed the manuscript outline and experimental framework. RG wrote the manuscript, designed and implemented the MaD python wrapper, and conducted the experiments. YC edited the manuscript and conducted the experiments. PS also conducted the experiments. AS contributed to the implementation of the MaD python wrapper and contributed to the figures in the manuscript. AB was involved in the discussion, experiment design, and editing of the manuscript. All authors contributed to the article and approved the submitted version.

## Conflict of Interest

The authors declare that the research was conducted in the absence of any commercial or financial relationships that could be construed as a potential conflict of interest.
