# Supplementary material for: HFNet: A CNN Architecture Co-designed for Neuromorphic Hardware With a Crossbar Array of Synapses
Source: Front Neurosci. 2020 Oct 26;14:907. doi: 10.3389/fnins.2020.00907 (PMC7649386; doi:10.3389/fnins.2020.00907)
Supplement: Supplementary file 1 [file Data_Sheet_1.PDF]

frontiers

Supplementary Material: HFNet: A CNN architecture co-designed for neuromorphic hardware with crossbar array of synapses

1 MAPPING AND DEBUGGING (MAD) FRAMEWORK OPTIMIZATIONS

1.1 Core Utilization

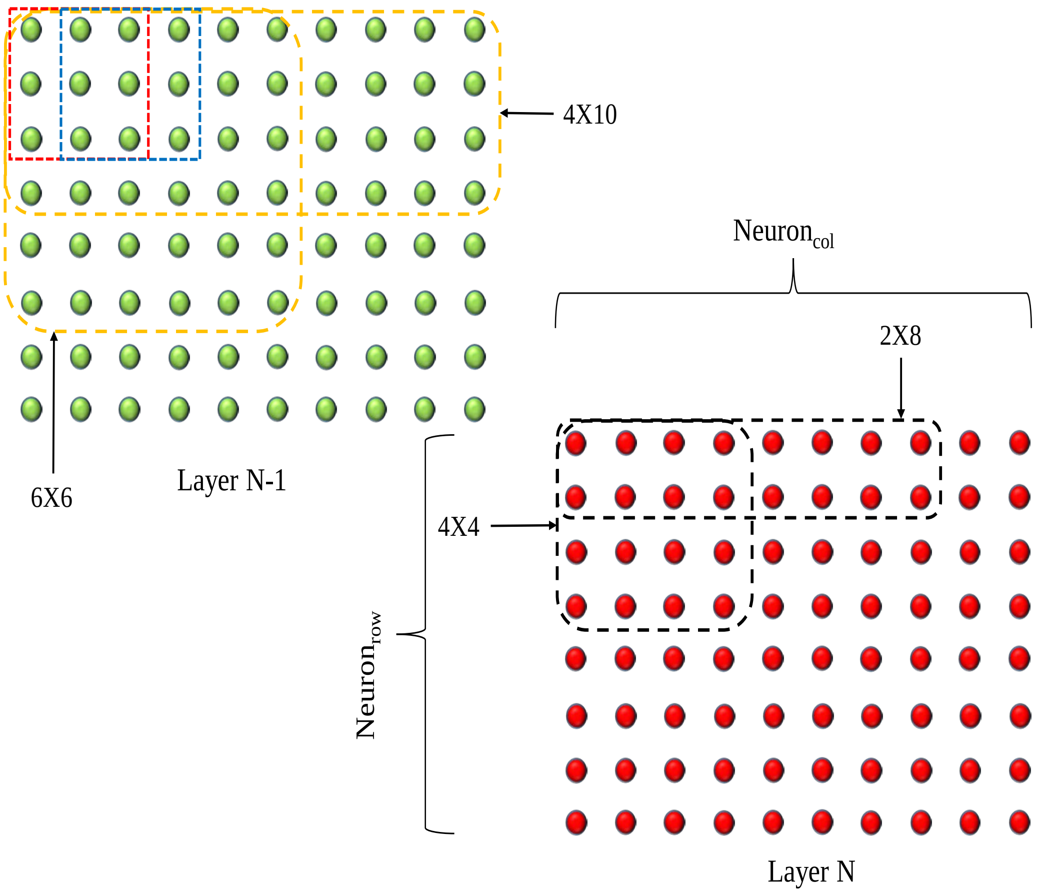

Figure S1. Illustration of the optimization of core utilization. Layer N-1 neurons are in green, whereas layer N neurons are in red. Synaptic connections are shown with dotted rectangular boxes in red and blue colour for two neurons in layer N. N_{axons} to be selected for 2 cases are shown in dotted rectangle in yellow colour in layer N-1.

Referring to fig. S1, consider a case for calculating core utilization, suppose 16 neurons are chosen from layer N for mapping onto a core. This can be done by choosing either 2 rows and 8 columns of neurons or 4 rows and 4 columns of neurons. Here, rows and columns of neurons correspond to $Neuron_{row}$ and $Neuron_{col}$ of main document. If the convolution kernel, K used is 3×3 and stride, S is 1, then for 2 rows and 8 columns of neurons the axons required are 4 rows and 10 columns, similarly for 4 rows and 4

columns of neurons the axons required are 6 rows and 6 columns. The rows and columns of axons required can be obtained as below:

$$O_{size} = \frac{I_{size} - K}{S} + 1 \tag{S1}$$

Where,
O_{size} = Size of the convolution output
I_{size} = Input size
K = convolution filter size
S = stride

Table S1. Input size calculation for different kernel sizes and strides

**Kernel Size, Stride**	**Input size for**	
	**Output size = 2×8**	**Output size = 4×4**
**2×2, 1**	3×9	5×5
**2×2, 2**	4×16	8×8
**3×3, 1**	4×10	6×6
**3×3, 2**	5×17	9×9
**4×4, 1**	5×11	7×7
**4×4, 2**	6×18	10×10
**5×5, 1**	6×12	8×8
**5×5, 2**	7×19	11×11

In the above example, one observes that choosing 4 rows by 4 columns of neurons is better for core utilization than choosing 2 rows and 8 columns as the input number of axons in the former is only 36 whereas in the latter, it is 40. Hence, core utilization is [36×16] in the former and [40×16] in the latter. The intuition from this example is that the neurons to be selected for mapping onto a core is better to be in a square shape rather than a rectangular shape. Table S1 gives an illustration of calculation of input size for different kernel sizes, strides in the two cases of output size of neuron selection (2×8 and 4×4). Here, we have only considered the case of filter sizes with equal width and height. Other filter shapes may not be directly relevant for CNN. It can be seen from the table that, the axon size is always less for the case of output size = 4×4 i.e. in square shape.

2 SUPPLEMENTARY TABLES

Table S2. HFNet-V3 with pooling and with fully connected layers

NN archi. Layers	HFNet model					
	HFNet-V3		*with pooling*		*with FC*	
	**Input Size**	**Output Size**	**Input Size**	**Output Size**	**Input Size**	**Output Size**
**C**[a]	226×226×3	112×112×32	226×226×3	112×112×32	226×226×3	112×112×32
**C**	114×114×32	56×56×64	114×114×32	112×112×64	114×114×32	56×56×64
**Pool**[b]			112×112×64	56×56×64		
**C**	58×58×64	28×28×256	58×58×64	28×28×256	58×58×64	28×28×256
**Pool**			56×56×256	28×28×256		
**D**[c]	30×30×256	28×28×256	30×30×256	28×28×256	30×30×256	28×28×256
**P**[d]	28×28×256	28×28×256	28×28×256	28×28×256	28×28×256	28×28×256
**D**	30×30×256	14×14×256	30×30×256	14×14×256	30×30×256	14×14×256
**P**	14×14×256	14×14×512	14×14×256	14×14×512	14×14×256	14×14×512
**D**	16×16×512	14×14×512	16×16×512	14×14×512	16×16×512	14×14×512
**P**	14×14×512	14×14×512	14×14×512	14×14×512	14×14×512	14×14×512
**D**	16×16×512	14×14×512	16×16×512	14×14×512	16×16×512	14×14×512
**P**	14×14×512	14×14×1024	14×14×512	14×14×1024	14×14×512	14×14×1024
**D**	16×16×1024	14×14×1024	16×16×1024	14×14×1024	16×16×1024	14×14×1024
**P**	14×14×1024	14×14×1024	14×14×1024	14×14×1024	14×14×1024	14×14×1024
**D**	16×16×1024	14×14×1024	16×16×1024	14×14×1024	16×16×1024	14×14×1024
**P**	14×14×1024	14×14×1024	14×14×1024	14×14×1024	14×14×1024	14×14×1024
**D**	16×16×1024	14×14×1024	16×16×1024	14×14×1024	16×16×1024	14×14×1024
**P**	14×14×1024	14×14×1024	14×14×1024	14×14×1024	14×14×1024	14×14×1024
**D**	16×16×1024	7×7×1024	16×16×1024	7×7×1024	16×16×1024	7×7×1024
**P**	7×7×1024	7×7×1024	7×7×1024	7×7×1024	7×7×1024	7×7×1024
**D**	9×9×1024	7×7×1024	9×9×1024	7×7×1024	9×9×1024	7×7×1024
**P**	7×7×1024	7×7×1000	7×7×1024	7×7×1000	7×7×1024	7×7×1024
**GAP**[e]	7×7×1000	1×1×1000	7×7×1000	1×1×1000	7×7×1024	1×1×1024
**FC**[f]					1×1×1024	1×1×1000

[a]Convolution layer.
[b]Max pooling layer.
[c]Depthwise convolution layer.
[d]Pointwise convolution layer.
[e]Global average pooling.
[f]Fully connected layer.

Table S3. Addition of layers to HFNet-V3

NN archi. Layers	HFNet model					
	HFNet-V3-M0		*HFNet-V3-M1*		*HFNet-V3-M2*	
	**Input Size**	**Output Size**	**Input Size**	**Output Size**	**Input Size**	**Output Size**
**C**[a]	226×226×3	112×112×32	226×226×3	112×112×32	226×226×3	112×112×32
**C**	114×114×32	56×56×64	114×114×32	112×112×64	114×114×32	112×112×64
**C**			114×114×64	56×56×112	114×114×64	56×56×112
**C**	58×58×112	28×28×256	58×58×112	28×28×256	58×58×112	28×28×256
**D**[b]	30×30×256	28×28×256	30×30×256	28×28×256	30×30×256	28×28×256
**P**[c]	28×28×256	28×28×256	28×28×256	28×28×256	28×28×256	28×28×256
**D**	30×30×256	14×14×256	30×30×256	14×14×256	30×30×256	14×14×256
**P**	14×14×256	14×14×512	14×14×256	14×14×512	14×14×256	14×14×512
**D**	16×16×512	14×14×512	16×16×512	14×14×512	16×16×512	14×14×512
**P**	14×14×512	14×14×512	14×14×512	14×14×512	14×14×512	14×14×512
**D**	16×16×512	14×14×512	16×16×512	14×14×512	16×16×512	14×14×512
**P**	14×14×512	14×14×1024	14×14×512	14×14×1024	14×14×512	14×14×1024
**D**	16×16×1024	14×14×1024	16×16×1024	14×14×1024	16×16×1024	14×14×1024
**P**	14×14×1024	14×14×1024	14×14×1024	14×14×1024	14×14×1024	14×14×1024
**D**	16×16×1024	14×14×1024	16×16×1024	14×14×1024	16×16×1024	14×14×1024
**P**	14×14×1024	14×14×1024	14×14×1024	14×14×1024	14×14×1024	14×14×1024
**D**	16×16×1024	14×14×1024	16×16×1024	14×14×1024	16×16×1024	14×14×1024
**P**	14×14×1024	14×14×1024	14×14×1024	14×14×1024	14×14×1024	14×14×1024
**D**	16×16×1024	7×7×1024	16×16×1024	7×7×1024	16×16×1024	7×7×1024
**P**	7×7×1024	7×7×1024	7×7×1024	7×7×1024	7×7×1024	7×7×1024
**D**	9×9×1024	7×7×1024	9×9×1024	7×7×1024	9×9×1024	7×7×1024
**P**	7×7×1024	7×7×1024	7×7×1024	7×7×1000	7×7×1024	7×7×1024
**D**	9×9×1024	7×7×1024			9×9×1024	7×7×1024
**P**	7×7×1024	7×7×1000			7×7×1024	7×7×1000
**GAP**[d]	7×7×1000	1×1×1000	7×7×1000	1×1×1000	7×7×1000	1×1×1000

[a]Convolution layer.
[b]Depthwise convolution layer.
[c]Pointwise convolution layer.
[d]Global average pooling.

Table S4. HFNet-V2-M0 and HFNet-V3-M3

NN archi. Layers	HFNet model			
	HFNet-V2-M0		*HFNet-V3-M3*	
	**Input Size**	**Output Size**	**Input Size**	**Output Size**
**C**[a]	226×226×3	112×112×32	226×226×3	112×112×32
**C**	114×114×32	56×56×56	114×114×32	112×112×64
**C**	58×58×56	28×28×256	114×114×64	112×112×112
**C**			114×114×112	56×56×112
**C**			58×58×112	28×28×256
**D**[b]	30×30×256	28×28×256	30×30×256	28×28×256
**P**[c]	28×28×256	28×28×512	28×28×256	28×28×256
**D**	30×30×256	14×14×512	30×30×256	14×14×256
**P**	14×14×256	14×14×512	14×14×256	14×14×512
**D**	16×16×512	14×14×512	16×16×512	14×14×512
**P**	14×14×512	14×14×512	14×14×512	14×14×512
**D**	16×16×512	14×14×512	16×16×512	14×14×512
**P**	14×14×512	14×14×512	14×14×512	14×14×1024
**D**	16×16×512	14×14×512	16×16×1024	14×14×1024
**P**	14×14×512	14×14×512	14×14×1024	14×14×1024
**D**	16×16×512	14×14×512	16×16×1024	14×14×1024
**P**	14×14×512	14×14×512	14×14×1024	14×14×1024
**D**	16×16×512	14×14×512	16×16×1024	14×14×1024
**P**	14×14×512	14×14×1024	14×14×1024	14×14×1024
**D**	16×16×1024	7×7×1024	16×16×1024	7×7×1024
**P**	7×7×1024	7×7×1000	7×7×1024	7×7×1024
**D**	9×9×1000	7×7×1000	9×9×1024	7×7×1024
**P**	7×7×1000	7×7×1000	7×7×1024	7×7×1000
**GAP**[d]	7×7×1000	1×1×1000	7×7×1000	1×1×1000

[a]Convolution layer.
[b]Depthwise convolution layer.
[c]Pointwise convolution layer.
[d]Global average pooling.

Table S5. HF-MobileNet models

NN archi. Layers	HF-MobileNet models			
	HF-MobileNet-V1		*HF-MobileNet-V2*	
	**Input Size**	**Output Size**	**Input Size**	**Output Size**
**C**[a]	226×226×3	112×112×32	226×226×3	112×112×32
**D**[b]	114×114×32	112×112×32	114×114×32	112×112×32
**P**[c]	112×112×32	112×112×32	112×112×32	112×112×64
**D**	114×114×64	56×56×64	114×114×64	56×56×64
**P**	56×56×64	56×56×128	56×56×64	56×56×128
**D**	58×58×128	56×56×128	58×58×64	56×56×128
**P**	56×56×128	28×28×128	56×56×128	28×28×128
**D**	30×30×128	28×28×128	30×30×128	28×28×128
**P**	28×28×128	28×28×256	28×28×128	28×28×256
**D**	30×30×256	28×28×256	30×30×256	28×28×256
**P**	28×28×256	28×28×256	28×28×256	28×28×256
**D**	30×30×256	14×14×256	30×30×256	14×14×256
**P**	14×14×256	14×14×512	14×14×256	14×14×256
**D**	16×16×512	14×14×512	16×16×256	14×14×256
**P**	14×14×512	14×14×512	14×14×256	14×14×512
**D**	16×16×512	14×14×512	16×16×512	14×14×512
**P**	14×14×512	14×14×512	14×14×512	14×14×512
**D**	16×16×512	14×14×512	16×16×512	14×14×512
**P**	14×14×512	14×14×1024	14×14×512	14×14×1024
**D**	16×16×1024	14×14×1024	16×16×1024	14×14×1024
**P**	14×14×1024	14×14×1024	14×14×1024	14×14×1024
**D**	16×16×1024	14×14×1024	16×16×1024	14×14×1024
**P**	14×14×1024	14×14×1024	14×14×1024	14×14×1024
**D**	16×16×1024	7×7×1024	16×16×1024	7×7×1024
**P**	7×7×1024	7×7×1024	7×7×1024	7×7×1024
**D**	9×9×1024	7×7×1024	9×9×1024	7×7×1024
**P**	7×7×1024	7×7×1024	7×7×1024	7×7×1024
**GAP**[d]	7×7×1024	1×1×1024	7×7×1024	1×1×1024
**FC**[e]	1×1×1024	1×1×1000	1×1×1024	1×1×1000

[a]Convolution layer.
[b]Depthwise convolution layer.
[c]Pointwise convolution layer.
[d]Global average pooling.
[e]Fully connected layer.

Table S6. HFNet-GC

NN archi. Layers	HFNet-GC	
	**Input Size**	**Output Size**
**C**[a]	$226\times226\times3$	$112\times112\times32$
**C**	$114\times114\times32$	$112\times112\times64$
**C**	$114\times114\times64$	$56\times56\times112$
**C**	$58\times58\times112$	$28\times28\times256$
**G**[b]	$30\times30\times256(8)$	$28\times28\times256(8)$
**G**	$30\times30\times256(8)$	$14\times14\times512(8)$
**G**	$16\times16\times512(8)$	$14\times14\times512(8)$
**G**	$16\times16\times512(8)$	$14\times14\times896(8)$
**G**	$16\times16\times896(8)$	$14\times14\times896(8)$
**G**	$16\times16\times896(8)$	$14\times14\times896(8)$
**G**	$16\times16\times896(8)$	$14\times14\times896(8)$
**G**	$16\times16\times896(8)$	$7\times7\times896(8)$
**G**	$9\times9\times896(8)$	$7\times7\times896(8)$
**G**	$9\times9\times896(8)$	$7\times7\times1000(8)$
**GAP**[c]	$7\times7\times1000$	$1\times1\times1000$

[a]Convolution layer.
[b]Grouped convolution layer.
Note: Number of groups are mentioned in the bracket.
[c]Global average pooling.

Table S7. Details of core utilization for HFNet-V3

HFNet-V3 Layers	Input Size	Output Size	Core utilization	Number of cores
**C**[a]	226×226×3	112×112×32	[459×1024]	392
**C**	114×114×32	56×56×64	[800×256]	784
**C**	58×58×64	28×28×256	[960×512]	392
**D**[b]	30×30×256	28×28×256	[900×784]	256
**P**[c]	28×28×256	28×28×256	[1024×1024]	196
**D**	28×28×256	14×14×256	[841×196]	256
**P**	14×14×256	14×14×512	[512×1024]	98
**D**	16×16×512	14×14×512	[1024×784]	128
**P**	14×14×512	14×14×512	[1024×1024]	98
**D**	16×16×512	14×14×512	[1024×784]	128
**P**	14×14×512	14×14×1024	[512×1024]	196
**D**	16×16×1024	14×14×1024	[1024×784]	256
**P**	14×14×1024	14×14×1024	[1024×1024]	196
**D**	16×16×1024	14×14×1024	[1024×784]	256
**P**	14×14×1024	14×14×1024	[1024×1024]	196
**D**	16×16×1024	14×14×1024	[1024×784]	256
**P**	14×14×1024	14×14×1024	[1024×1024]	196
**D**	16×16×1024	7×7×1024	[900×196]	256
**P**	7×7×1024	7×7×1024	[1024×1024]	49
**D**	9×9×1024	7×7×1024	[972×588]	86
**P**	7×7×1024	7×7×1000	[1024×1000]	49

[a]Convolution layer.
[b]Depthwise convolution layer.
[c]Pointwise convolution layer.
[d]Global average pooling.

Table S8. Details of core utilization for MobileNet

MobileNet Layers	Input Size	Output Size	Core utilization	Number of cores
**C**[a]	224×224×3	112×112×32	[459×1024]	392
**D**[b]	112×112×32	112×112×32	[1024×900]	512
**P**[c]	112×112×32	112×112×64	[512×1024]	784
**D**	112×112×64	56×56×64	[1023×240]	1024
**P**	56×56×64	56×56×128	[512×1024]	392
**D**	56×56×128	56×56×128	[1024×900]	512
**P**	56×56×128	56×56×128	[1024×1024]	392
**D**	56×56×128	28×28×128	[1023×240]	512
**P**	28×28×128	28×28×256	[512×1024]	196
**D**	28×28×256	28×28×256	[900×784]	256
**P**	28×28×256	28×28×256	[1024×1024]	196
**D**	28×28×256	14×14×256	[841×196]	256
**P**	14×14×256	14×14×512	[512×1024]	98
**D**	14×14×512	14×14×512	[1024×784]	128
**P**	14×14×512	14×14×512	[1024×1024]	98
**D**	14×14×512	14×14×512	[1024×784]	128
**P**	14×14×512	14×14×512	[1024×1024]	98
**D**	14×14×512	14×14×512	[1024×784]	128
**P**	14×14×512	14×14×512	[1024×1024]	98
**D**	14×14×512	14×14×512	[1024×784]	128
**P**	14×14×512	14×14×512	[1024×1024]	98
**D**	14×14×512	14×14×512	[1024×784]	128
**P**	14×14×512	14×14×512	[1024×1024]	98
**D**	14×14×512	7×7×512	[900×196]	128
**P**	7×7×512	7×7×1024	[512×1024]	49
**D**	7×7×1024	7×7×1024	[972×588]	86
**P**	7×7×1024	7×7×1024	[1024×1024]	49

[a]Convolution layer.
[b]Depthwise convolution layer.
[c]Pointwise convolution layer.
